# Phenotypic and epigenetic profiles of circulating NK cells in spontaneous HIV-1 controllers

**DOI:** 10.1016/j.ebiom.2025.105948

**Published:** 2025-09-29

**Authors:** Alisa Huber, Albert L. Groenendijk, Adriana Navas, Nadira Vadaq, Suzanne D.E. Ruijten, Vasiliki Matzaraki, Ezio T. Fok, Aysel Gurbanova, Wilhelm A.J.W. Vos, Marc J.T. Blaauw, Louise E. van Eekeren, Maartje C.P. Jacobs-Cleophas, Janneke Stalenhoef, Marvin Berrevoets, Renate van der Molen, Arnold van der Meer, Marien I. de Jonge, Joost H.A. Martens, Casper Rokx, Annelies Verbon, Jan van Lunzen, Hans J.P.M. Koenen, Mihai G. Netea, Andre J.A.M. van der Ven, Leo A.B. Joosten, Jéssica C. dos Santos

**Affiliations:** a Department of Internal Medicine and Radboudumc Community for Infectious Diseases (RCI), Radboudumc, Nijmegen, the Netherlands; b Department of Internal Medicine and Department of Medical Microbiology and Infectious Diseases, ErasmusMC, Erasmus University, Rotterdam, the Netherlands; c Department of Cell Biology, Faculty of Science, Radboud University, Nijmegen, the Netherlands; d Laboratory of Medical Immunology (LMI), Radboudumc, Nijmegen, the Netherlands; e Department of Internal Medicine and Infectious Diseases, OLVG, Amsterdam, the Netherlands; f Department of Internal Medicine and Infectious Diseases, Elizabeth-Tweesteden Ziekenhuis, Tilburg, the Netherlands; g Department of Immunology and Metabolism, Life and Medical Sciences Institute, University of Bonn, Germany; h Department of Medical Genetics, Iuliu Hatieganu University of Medicine and Pharmacy, Cluj-Napoca, Romania

**Keywords:** NK-cells, HIV, Innate immune memory, Spontaneous HIV-controllers, Normal progressors on ART

## Abstract

**Background:**

NK cells play a key role in eliminating HIV-infected cells, but it is unclear whether there are specific NK cell receptor signatures in spontaneous HIV controllers.

**Methods:**

We conducted a cross-sectional analysis of circulating NK cell phenotypes in people living with HIV (PLHIV), divided into spontaneous HIV controllers (HIC), normal progressors on antiretroviral therapy (non-HIC), and first-degree HIV-negative family members. Using supervised and unsupervised flow cytometry, we assessed NK cell markers and receptors. We performed an epigenetic analysis of H3K4me3 chromatin enrichment in NK cells from both HIC and non-HIC and measured IFNγ, Perforin and CD107a expression in NK cells upon stimulation with IL-2/IL-15, K562 cells, and IFNα. Additionally, we conducted a genome-wide association study (GWAS) and quantitative trait locus (QTL) mapping using data from HIC and non-HIC part of the 2000HIV study.

**Findings:**

HIV controllers had higher levels of CD56^bright^ NK cells and increased expression of NKp46, NKp30, and DNAM-1. The genetic association between protective MHC class I alleles and the NK cell receptor KIR2DL2/3 supports a genetic predisposition to HIV control. Unsupervised clustering identified an HIV-induced NK cell population, separate from CMV-induced NK cells. Epigenetic analysis revealed greater H3K4me3 marks in genes involved in immune response pathways, including IFNα, IL-15, and IL-2. The memory-like NK cell subpopulation was characterised by elevated expression of NKG2C and ILT2, with reduced KIR2DL2/3 in HIC. These memory NK cells were more responsive to stimulation with IFNα, resulting in increased production of IFNγ in HIC.

**Interpretation:**

These results suggest that spontaneous HIV control is associated with an NK cell memory phenotype, shaped by HIV infection, epigenetic modifications, and genetic factors.

**Funding:**

The authors are part of the 2000HIV study, which is supported by ViiV Healthcare.

**Research in context**

**Evidence before this study**

Spontaneous HIV controllers are a rare subset of PLHIV who can control the virus without antiretroviral therapy (ART). The mechanisms enabling this control remain incompletely understood. NK cells, key players in the innate immune system, are recognised for their ability to mediate antiviral responses and have been implicated in HIV control. Sources included PubMed, Scopus, and Web of Science databases, as well as reference lists from key journal articles and books. The search was conducted from 2021 to November 2024 using the keywords “HIV controllers”, “NKG2C”, “memory-like NK cells”, and “HIV innate immunity”. We identified a total of 13 studies that mention memory-like NK cells in HIV or SIV and suggest that NK cells, especially those expressing NKG2C, may be involved in modulating viral load, with some studies reporting a correlation between NKG2C^+^ NK cells and viral set points. However, the role of genetic and epigenetic factors in shaping NK cell responses in HIV controllers and the distinction between HIV- and CMV-induced memory NK cells remain unclear.

**Added value of this study**

This study identifies a subset of memory-like NK cells associated with spontaneous HIV control, providing new insights into the immune mechanisms underlying HIV suppression. HIV-induced memory NK cells that were identified in this study show distinct receptor profiles and functional properties, with genetic and epigenetic factors playing a role in their development.

**Implications of all the available evidence**

The identification of HIV-specific memory-like NK cells expands our understanding of innate immune responses in HIV control, distinguishing them from CMV-induced NK cells. These findings highlight the potential for targeting NK cell pathways in therapeutic interventions and suggest that genetic and epigenetic factors may be important for developing personalised strategies for HIV treatment and prevention. Future studies could use this identified NK cell phenotype to isolate and expand memory NK cells ex vivo, offering new opportunities for personalised therapies and immune-based treatments for HIV.

## Introduction

Human immunodeficiency virus (HIV) type 1 is spontaneously controlled in the absence of antiretroviral therapy (ART) by a small percentage of people living with HIV (PLHIV), known as HIV controllers.^1^ Studies have shown that the adaptive immune system plays a central role in HIV control, specifically the antigen-specific responses of CD8^+^ T cells through interaction with major histocompatibility complex (MHC) class I molecules.^2^^,^^3^ Natural killer (NK) cells, effector cells of the innate immune system, share functional similarities with CD8^+^ T cells, including their ability to interact with MHC molecules, cytotoxic activities, and secretion of activating cytokines. Unlike CD8^+^ T cells, NK cells contribute to the early containment of HIV replication and can exert their effects in an antigen-independent manner.^4^, ^5^, ^6^

NK cells originate from a common CD34+ lymphoid progenitor in the bone marrow, which is subsequently differentiated into distinct subpopulations of peripheral circulating NK cells based on the density of CD56 and CD16 on the cell surface. Circulating NK cells are classified into three main subsets: CD56^bright^ NK cells (CD56^bright^ CD16^−^ and CD56^bright^ CD16^+^) make up around 10% of peripheral blood NK cells. They are less mature and exhibit high proliferative capacity towards cytokines such as IL-2 and IL-15, and primarily function as cytokine producers.^7^ They contain low levels of perforin and granzyme B and are enriched in secondary lymphoid tissue. CD56^dim^ cells (CD56^dim^ CD16^−^ and CD56^dim^ CD16^+^) comprise around 90% of circulating NK cells and represent the mature cytotoxic effector population. They express high levels of perforin and granzyme B, as well as activating receptors (NKG2D, DNAM-1, and natural cytotoxicity receptors) as well as inhibitory receptors (KIRs, NKG2A).^8^^,^^9^ Their maturation involves downregulation of CD56 and NKG2A, acquisition of CD16 and KIRs, and ultimately expression of CD57, marking terminal differentiation.^8^^,^^9^ CD56^low^ NK cells (CD56^low^ CD16^+^), an additional subset, which is found in chronic viral infections and often referred to as dysfunctional NK cells.^10^^,^^11^ The increase in CD16 expression and the subsequent decrease in CD56 expression correlate with NK cell maturation.^10^^,^^12^ The functionality of NK cells is intricately regulated by a diverse array of activating and inhibitory receptors. Based on the NK cell receptor repertoire present in the membrane, distinct functional NK cell subpopulations are identified. NKp30 and NKp46 are known as natural cytotoxicity receptors (NCRs) that mediate cytotoxic effects, and their expression has been associated with a relatively smaller HIV reservoir size.^13^^,^^14^ Other NK cell receptors, such as DNAM-1 and NKG2D, are essential for NK cell activation and activating stress responses, respectively.^15^^,^^16^ The inhibitory receptors, such as KIR2DL2/3, ILT2 (LILRB1), and NKG2A, play a role in modulating immune activation by downregulating NK cell effector responses.^17^ Importantly, Killer Immunoglobulin-Like Receptors (KIRs) interact with MHC class-I molecules^18^ and have been implicated in HIV control.^19^, ^20^, ^21^, ^22^, ^23^ CD57+ NK cells indicate a functionally distinct, mature NK cell population and have been shown to mediate robust antibody-mediated cytotoxicity (ADCC) against HIV-infected cells. Additionally, it defines a population of highly differentiated or classical fully mature NK cells characterised by the KIR+LIR-1+NKG2A−NCR± phenotype.^24^, ^25^, ^26^

Memory-like NK cells have been identified in the context of viral infections such as cytomegalovirus (CMV), Hantavirus, Chikungunya virus, and upon exposure to the combination of cytokines IL-12, IL-15, and IL-18.^27^, ^28^, ^29^, ^30^ An additional phenotype of memory-like NK cells, named pregnancy-trained decidual NK cells, has been identified in repeated pregnancies.^31^ Memory-like NK cells are long-lived innate cells that exhibit enhanced recall responses during subsequent reactivation. The expansion of memory-like NK cell population among PLHIV due to the frequent co-infection with CMV is documented, which may be important for the NK cell repertoire, as CMV-HIV co-infection has a high prevalence, reaching 95%, particularly in men having sex with men (MSM).^32^ In humans, memory-like NK cells from CMV-seropositive individuals are characterised by the expression of CD94, NKG2C, CD57, and increased production of IFNγ.^31^ Previous research indicates that CD57+ NKG2C+ NK cells can mount effective immune responses mediated by broad neutralising antibodies, suggesting their critical role in controlling HIV viraemia.^25^^,^^26^ These NK cells also exhibit transcriptional profiles associated with heightened ADCC capability, further underscoring their functional importance in HIV pathogenesis. In CMV infection, the presentation of viral peptides (such as UL40-derived peptides) by human leucocyte antigen E (HLA-E) drives the activation and expansion of adaptive NKG2C+ NK cells, even when classical MHC class I molecules are downregulated.^33^^,^^34^ A parallel HLA-E/NKG2C-mediated pathway operates in HIV infection, whereby HIV-derived peptides presented by HLA-E engage NKG2C^+^ NK cells and drive their cytotoxic expansion, mirroring the adaptive response seen in CMV.^35^^,^^36^ These findings highlight the role of HLA-E/NKG2C interactions in shaping NK cell responses in both infections. Notably, the robust activation of memory NK cells not only aids in controlling CMV but also sets the groundwork for improved responsiveness to other viral infections by enhancing memory NK cell pools. However, the receptor signature of NK cells that expand in HIV or CMV co-infections, as well as their implications for spontaneous HIV control, are only partially understood.

The present study aimed to characterise the NK cell signature from HIV controllers by whole-blood immunophenotyping. We study a cohort of 92 HIV controllers (HIC) and a large group of 1006 PLHIV on long-term ART (non-HIC). Results from PLHIV were compared to healthy controls, which were composed of HIV-negative first-degree family members of both HIC and non-HIC. First-degree family members were used as healthy controls and proxies to understand the NK cell repertoire before HIV infection and to limit the influence of genetically pre-determined NK cell functions. We analysed the overall composition and phenotype of circulating NK cells by assessing markers of activation, exhaustion, and chemokine receptors. In addition, we assessed the expression of functional activating and inhibitory receptors on CD56-sorted NK cells from a subset of HIC, non-HIC, and family members. Our results demonstrate that NK cell composition differs between HIV controllers and non-controllers. Additionally, we identified a unique subset of NK cells presenting memory-like features as a hallmark of HIV control. These data were combined with MHC class I alleles, which were previously identified in spontaneous HIV controllers. Based on these analyses, we conclude that single nucleotide polymorphisms (SNPs) located in the MHC class I locus, known to be associated with spontaneous HIV control, may contribute to the NK cell characteristics identified in HIV controllers.

## Methods

### Cohort

Participants were enrolled between October 2019 and October 2021 in the 2000HIV Human Functional Genomics Partnership Program (2000HIV study, NCT03994835), a study including 1895 people living with HIV-1 (PLHIV), representing approximately 10% of PLHIV in the Netherlands, as described elsewhere.^37^ In short, the inclusion criteria were proven HIV infection in subjects aged 18 years or older, cART exposure for at least six months, and having a most recent HIV-1 RNA load of less than 200 copies/mL. Exclusion criteria were current pregnancy, detectable viral hepatitis B or C DNA, as well as signs of any current acute infection. HIC was defined as individuals with a history of spontaneous control of HIV-1 with viral loads lower than <10,000 copies/mL for at least five years, during which CD4^+^ T-cell counts were stable >500 cells/mm^3^.^37^ Some of the included HIC were not on cART (n = 20) but were able to maintain a stable viral load and meet the above-described criteria to be included in the study and defined as spontaneous HIV controller. ART was initiated in the remaining HIV controllers (n = 72) as new HIV treatment guidelines were introduced in 2015, which recommended ART for all PLHIV regardless of viral load levels or CD4 counts. Both ART-naïve and ART-exposed HIC are summarised as HIC in this study. The 2000HIV study is composed of discovery and validation cohorts, of which the participants of the discovery cohort were recruited from three specialised Dutch HIV treatment centres, two university medical centres, and one large general hospital (Radboudumc Nijmegen, Erasmus MC Rotterdam, and OLVG Amsterdam). Flow cytometry data were analysed from 1098 PLHIV, all from the 2000HIV discovery cohort.

The 2000HIV-TRAINED substudy (NCT04968717) enrolled PLHIV, including HIV controllers (HIC, n = 31), non-controllers on cART (non-HIC, n = 30) and HIV-1 negative 1st-degree family members of PLHIV (n = 43) referred to as healthy controls (HC) hereafter. All participants were included between August and October 2021. The exclusion criteria for the family members were the presence of inflammatory comorbidities, or use of immunomodulating medication, or positive serology for HIV-1. All participants were excluded in case of a recent infection or vaccination (<4 weeks) prior to the date of inclusion. Apart from being HIV negative, the inclusion criteria for the family members were comparable to those of PLHIV. The definition of being a spontaneous HIV controller was the same as in the 2000HIV substudy.

### Ethics

All study participants provided written informed consent. The 2000HIV study and the 2000HIV-TRAINED substudy were approved by the Medical Ethical Review Committee Oost Nederland, Nijmegen, the Netherlands, NL68056·091·81 and NL76999·091·21, respectively.

### Flowcytometry

#### NK cell phenotyping on whole-blood

Whole blood was obtained from venous blood collected in EDTA tubes, and NK cell subsets were assessed by flowcytometry as previously described by Navas et al.^38^ NK cell phenotyping was done in 1098 PLHIV, including 92 HIC and 1006 non-HIC, as well as in 43 healthy controls, using two panels. Healthy controls were from the 2000HIV-TRAINED substudy. CD56 and CD16 expression in 5 subpopulations of NK cells were identified as follows: CD56^bright^CD16^−^, CD56^bright^CD16^+^, CD56^dim^CD16^+^, CD56^dim^CD16^−^, and CD56^low^CD16^+^ using antibodies from Panel 1 (Tables S1 and S2). Activating and exhaustion markers, as well as chemokine receptors (PD1, CD38, HLA-DR, CXCR3, and CXCR5), were measured on total NK cells in Panel 2. For this study, we used DuraClone tubes by Beckman Coulter, which were custom-made. Table S1 shows the information for all drop-in antibodies of Panel 1 and Panel 2; the remaining antibodies (CXCR3-AF448, CD16-FITC, CD45-Kro, CD19-AA700, CD56-ECD, CD123-PC7, and PD1-PC5·5) were pre-coated in the DuraClone tubes (Table S2). All antibodies and cell lines used in this study were commercially sourced and validated by the respective manufacturers. Detailed information, including target, clone, and catalogue number, is provided in the relevant Methods sections.

### Genome-wide association study on HIV control

A GWAS was performed in a cohort of 67 HIV controllers and 1179 non-controllers of European ancestry. A logistic regression model was used in which age, sex and the first five genetic PCs, to correct for population stratification, were included as confounders. Potential confounding variables were chosen a priori using the modified disjunctive cause criterion, which recommends adjusting for any measured variable that is a plausible cause of the exposure, the outcome, or both, while avoiding colliders and pure instruments.^39^ Association testing was performed using PLINK v1·90b. Suggestive SNPs (p < 1 × 10^−5^) were greedily clumped using PLINK 1·90b with an r^2^ threshold of 0·1 and a distance threshold of 500 kb.

### Quantitative trait locus (QTL) mapping

Using both the 2000HIV-study discovery and validation cohorts, we performed QTL mapping for PBMC gene expression (n_disc_ = 1048, n_val_ = 260) and plasma protein concentrations (n_disc_ = 1064, n_val_ = 266) as described by Botey-Bataller et al.^40^ In short, QTL mapping was performed using the MatrixEQTL package,^41^ using a linear model with inverse-rank transformed gene expression or plasma protein concentrations, correcting for age, sex, BMI, seasonality, inclusion before the COVID pandemic, COVID vaccination, and inclusion centre. Functional effects of lead SNPs in the MHC region were prioritised using genome-wide significant QTLs (p < 5 × 10^−8^) in the discovery cohort, which could be validated in the validation cohort (p < 0·05, consistent directionality) for either the lead SNP or a proxy (r^2^ > 0·8).

### Assessment of functional NK cell markers on CD56_+_ isolated NK cells

Human peripheral blood mononuclear cells (PBMCs) of 23 PLHIV (13 HIC and 10 non-HIC) as well as from 21 healthy controls, were obtained from venous blood collected in EDTA tubes and were isolated through density centrifugation of blood diluted 1:1 in pyrogen-free phosphate-buffered saline (PBS) over Ficoll–Paque (GE Healthcare, UK).^37^ CD56^+^ NK cells were positively sorted from PBMCs through CD56 MicroBeads (Miltenyi Biotec, Bergisch Gladbach, Germany) and MACS cell-separation system (Miltenyi Biotec, Bergisch Gladbach, Germany) according to the manufacturer's protocol. 0·5 × 10^6^ cells were analysed on the twenty-one-colour, six-laser CytoFLEX-LX with Cytoexpert software 2·3 (Beckman Coulter, Brea, California, USA). The panel was focused on NK cell subpopulations and included ViaKrome-IR885/40 (#C36628), PacBlue-CD94 (clone HP-3B1, #B90465), KrO-CD45 (clone J33, #B36294, RRID: AB_2833027), FITC-CD57 (clone NC1, #IM0466U), PC5·5-CD158b1/b2,j (KIR2DL2/3; clone GL183, #A22333, RRID: AB_3662729), PC7-CD159a (NKG2A; clone Z199, #B10246, RRID: AB_2687887), APC-CD314 (NKG2D; clone ON-72, #A22329), APCA-A700-CD56 (clone N901, #IM2474U, RRID: AB_2876784), APCA-A750-CD3 (clone UCHT1, #A94680) (Beckmann Coulter, Brea, California, USA); BUV395-HLADR (clone G46-6, #564040, RRID: AB_2738558), BUV496-CD16 (clone 3G8, #612944, RRID: AB_2870224) (BD Biosciences, Franklin Lakes, New Jersey, USA); SNv605-CD337 (NKp30; clone P30-15, #325234), SNv650-CD335 (NKp46; clone 9 E2, #331927, RRID: AB_2562442), SNv785-CD226 (DNAM-1; clone 11A8, #338322, RRID: AB_2721560), ECD-CD85j (ILT2; clone GHI/75, #333708, RRID: AB_2136385) (BioLegend, San Diego, California); PE-NKG2C (clone 134591, #FAB138P-100, RRID: AB_2132983) (R&D Systems, Minneapolis, Minnesota, USA). Compensation matrices were constructed, and FMO controls were used to determine positive and negative populations for NKG2C, CD85j, CD94, CD158b1, CD159a, CD226, CD314, CD335, and CD337. The CD56^+^ isolates were gated on live singlets and CD45^+^CD56^+^CD3^−^ in Kaluza V2·1·3 (Kaluza, London, UK). The data was further exported and uploaded to the Cytobank platform (V9·0, Beckman Coulter, Brea, California, USA).

### Unsupervised flowcytometry analysis of CD56^+^CD3^−^ NK cells

Unsupervised analyses were performed on the Cytobank platform (V9·0, Beckman Coulter, Brea, California, USA) to identify relevant populations. As a dimension reduction map, we used Uniform Manifold Approximation and Projection (UMAP) to visualise the composition of the NK cell repertoire. Next, unsupervised clustering analysis was performed with FlowSOM (Flow cytometry Self-Organising Map) to identify NK cell metaclusters. The metaclustering method was ‘hierarchical consensus’ with equal sampling (29,969 events of clean-live CD45+CD3−CD56+ cells/sample for 44 individuals, after four individuals with <17,000 events were excluded) so that, in total, 1,318,636 events were included in the algorithm. Iterations and learning rate were set at automatic (879 and 109,886, respectively), perplexity = 30, early exaggeration = 12, the generated seed = 1,315,627,013, metaclusters = 15, and clusters = 100.

### CMV serology

All study participants were tested for anti-CMV IgG serology using an ELISA (DEIA326, Creative Diagnostics, Shirley, New York, US), according to the manufacturer's instructions.

### NK cell cytotoxicity assay and interferon-gamma production

Frozen PBMCs of 4 HIC and 4 non-HIC PLHIV were seeded at 500,000 cells per well and activated overnight with rhIL-2 (100 U/mL, Proleukin, Alloga, United Kingdom) and rhIL-15 (10 ng/mL, Gibco, Thermo Fisher Scientific, Carlsbad, California, USA). MHC-negative K562 cells (RRID: CVCL_K562) were labelled with 0·2 μM CFSE (BD Biosciences, Franklin Lakes, New Jersey, USA) overnight. After incubation, K562^CFSE^ cells were added to the PBMCs in a 1:10 ratio together with an anti-CD107a antibody (PE-CD170a, clone H4A3, #560948, BD Biosciences, Franklin Lakes, New Jersey, USA). Alternatively, rh-IFNα (100 U/mL, BioTechne, Minneapolis, Minnesota, USA) was added to the PBMCs. After 1 h of incubation at 37 °C, 60 μg/mL Brefeldin A (Sigma–Aldrich, Saint-Louis, Missouri, USA) was added to the co-culture and incubated for another 3 h. Afterwards, cells were stained for extracellular markers KrO-CD45 (clone J33, #B36294, RRID: AB_2833027), PC5·5-CD158b1/b2,j (KIR2DL2/3; clone GL18, #A22333, RRID: AB_3662729) (Beckmann Coulter, Brea, California, USA), BV605-CD3 (clone HIT3alpha, #564712, RRID: AB_2738908), BV711-CD56 (clone NCAM16·2, #664524, RRID: AB_2916895) (BD Biosciences, Franklin Lakes, New Jersey, USA), AF700-NKG2C (clone 134522, #FAB1381N-100UG, RRID: AB_3646367, R&D Systems, Minneapolis, Minnesota, USA), ECD-CD85j (ILT2; clone GHI/75, #333708, RRID: AB_2136385, BioLegend, San Diego, California) and intracellular markers, PB-Perforin (clone dG9, #308102, RRID: AB_314700) (BioLegend, San Diego, California) and PE-Cy7-IFNγ (clone 4s.B3, #25-7319-82, RRID: AB_469682, Thermo Fisher Scientific, Carlsbad, California, USA), as well as for viability (ViaKrome-IR885/40, #C36628, Beckmann Coulter, Brea, California, USA). To standardise cell count, 50 μL per sample CountBright Plus Absolute Counting Beads were used (Thermo Fisher Scientific, Carlsbad, California, USA). Marker expression was measured with the six-laser CytoFLEX-LX with Cytoexpert software 2·3 (Beckman Coulter, Brea, California, USA) and Kaluza V2·1·3 (Kaluza, London, UK).

### *Ex-vivo* cytokine production assay

0·5 × 10^6^ PBMCs/well from HIC (n = 29), non-HIC (n = 30) and HC (n = 41) of the 2000HIV-TRAINED study were seeded in U-bottom plates (Corning, Tewksbury, Massachusetts, USA) and subsequently stimulated with viral peptides HIV-Env (HIV envelope pool (1 μg/mL) JPT Peptide Solutions, Berlin, Germany) and pp65-CMV (1 μg/mL, JPT Peptide Solutions), and a Toll-like receptor (TLR)-7 ligand (imiquimod-IMQ (5 μg/mL), Invivogen, San Diego, California, USA) for 24 h at 37 °C and 5% CO_2_. The supernatant was collected and stored at −20 °C until used for cytokine measurements of IL-2, IL-12, IL-15, IL-18, and IFNα. Measurement was performed using an Ella Automated Immunoassay System (BioTechne, Minneapolis, Minnesota, USA) according to the manufacturer's recommendations. Statistical analysis was performed using the non-parametric Sign-test. For stimulation with CMV-pp65, only CMV-seropositive participants were included in the analysis.

### ChIP sequencing of CD56^+^ NK cells

CD56^+^ NK cells from the participants of the 2000HIV-TRAINED study were enriched using MACS kit as described above. H3K4me3 chromatin enrichment was assessed in 200,000 CD56^+^ NK cells of HIC (n = 8) and non-HIC (n = 5) (https://doi.org/10.34973/v0gn-8w71) through CUT&RUN method according to the manufacturer's recommendations (Cell Signalling, Danvers, MA, USA).^42^ Chromatin immunoprecipitation (ChIP)-seq libraries were prepared using the Kapa Hyper Prep Kit according to manufacturer's protocol, with the following modifications. 2·5 mL of the NEXTflex adaptor stock (600 nM, Bioo Scientific) was used for adaptor ligation of each sample. Libraries were amplified with 12–15 PCR cycles, followed by a double post-amplification clean-up to ensure proper removal of adaptors. Samples were analysed for purity using a High Sensitivity DNA Chip on a Bioanalyzer 2100 system (Agilent). Libraries were paired-end sequenced to a read length of 50 bp on an Illumina NextSeq500. ChIP-sequencing data was analysed using Seq2science.^43^ Briefly, raw sequencing reads were aligned to human genome hg38 with BWA.^44^ Samtools was used to filter reads with a quality score lower than 20, and PCR duplicates were removed with Picard.^45^ Peaks were identified with MACS 2·2·6 in paired-end mode and ‘call-summits’ enabled at a false discovery rate of 0·01.^46^ A union of all identified peaks was generated with BEDTools, which was used to count reads per peak in each sample.^47^ Significantly altered peaks were filtered using the following settings: Sum of average reads per peak >50 and fold difference of mean ± 2x stdev. Heatmap was based on row-normalised z-scores of significantly altered peaks using the ggplot2 R package. For pathway enrichment analysis, the clusterProfiler R package was used to analyse the Hallmark gene sets, with the nearest gene to each significantly altered peak serving as the input.

### Statistical analysis

Comparisons were tested for statistical significance with Wilcoxon rank-sum tests in R 4·3·1 (RStudio, PBC, Boston, MA, USA), and visualisations were made with GraphPad Prism (version 10·9·2 for Windows, GraphPad Software, Boston, MA, USA) and R 4·3·1. Group comparisons (Table 1 and Table 2) were conducted using appropriate statistical tests based on variable type and distribution. For continuous variables, Wilcoxon rank-sum test was used to compare means between two groups, accounting for potential unequal variances. Categorical variables were compared using the Chi-square test when expected cell counts were adequate (≥5). Fisher's exact test was applied for categorical comparisons when the expected frequency in any cell was less than 5. Statistical significance was set at p < 0·05. The relative percentages of NK cell populations from 1098 PLHIV, 43 HC, and 1006 non-HIC versus 92 HIC were normalised using an inverse rank transformation and compared with a linear regression model, corrected for sex, age, season of inclusion, time to laboratory processing, and COVID-19 vaccination, as these variables met the modified disjunctive cause criterion. The relative percentages of NK cell populations in CD56+ sorted NK cells from the 2000HIV TRAINED cohort, including n = 21 HC and n = 23 PLHIV, were normalised using an inverse rank transformation and compared with a linear regression model, corrected for sex and age, as they remained as plausible confounders under the same criterion. Covariates were selected based on regression with principal components and sensitivity analysis (Fig. S1). Regression predictors had 0% missingness, and only a small proportion of participants (<5·5%) had missing values in specific flow cytometry markers. Given this very low level of missingness and the large remaining sample size, complete-case analysis was used.

**Table 1:** Baseline characteristics of PLHIV, HIV controllers and non-controllers, part of the 2000HIV discovery cohort and healthy controls of the 2000HIV TRAINED-substudy of which whole blood flow cytometry analysis was performed.

Variable	HC^a^ n = 43	PLHIV^a^ n = 1120	p-value^b^	HIC^a^ n = 92	Non-HIC^a^ n = 1006	p-value^b^
**Sex**			1·5 × 10^−5^			0·045
Male	14 (32·6%)	929 (82·9%)		69 (75·0%)	843 (83·8%)	
Female	29 (67·4%)	191 (17·1%)		23 (25·0%)	163 (16·2%)	
**Age in years**	53·0 (15·9)	51·1 (12·0)	0·26	50·4 (11·9)	51·3 (11·9)	0·35
**Covid19 vaccination**			2·5 × 10^−4^			2·6 × 10^−4^
No	10 (23·3%)	736 (65·7%)		45 (48·9%)	677 (67·3%)	
Yes	33 (76·7%)	384 (34·3%)		47 (51·1%)	329 (32·7%)	
**Ethnicity**			0·11			0·006
Asian	0 (0·0%)	60 (5·4%)		4 (4·3%)	54 (5·4%)	
Black	2 (4·7%)	133 (11·9%)		12 (13·0%)	118 (11·7%)	
Hispanic	3 (7·0%)	40 (3·6%)		3 (3·3%)	36 (3·6%)	
Other	2 (4·7%)	101 (9·0%)		18 (19·6%)	81 (8·1%)	
White	36 (83·7%)	786 (70·2%)		55 (59·8%)	717 (71·3%)	
**CD4 latest in ×10^9^ cells/L**	NA	0·7 (0·5, 0·9)		0·7 (0·6, 1·0)	0·7 (0·5, 0·9)	0·023
**CD4 Nadir in ×10^9^ cells/L**	NA	0·3 (0·2)		0·5 (0·3, 0·6)	0·2 (0·1, 0·4)	5·3 × 10^−5^
**VL latest**						3·7 × 10^−4^
Undetectable^c^	NA	1085 (96·9%)		83 (90·2%)	980 (97·4%)	
Detectable^d^	NA	35 (3·1%)		9 (9·8%)	26 (2·6%)	
**VL latest in copies/mL (for detectable means)**	NA	50·0 (34·0, 78·0)		166·0 (42·0, 461·0)	43·0 (34·0, 63·0)	0·010
**HIV duration, years**	NA	13·2 (7·9, 20·0)		16·7 (7·9)	14·3 (8·3)	0·006
**ART usage**						3·2 × 10^−4^
Yes	NA	1095 (97·8%)		67 (72·8%)	1006 (100·0%)	
No	NA	25 (2·2%)		25 (27·2%)	0 (0·0%)	
**CMV serostatus**						0·81
Negative	7 (7·6%)	72 (6·5%)		7 (7·6%)	64 (6·4%)	
Positive	85 (92·4%)	1044 (93·5%)		85 (92·4%)	939 (93·6%)	
Unknown	0	4		0	3	

HIC, HIV controllers; Non-HIC, normal progressors PLHIV on suppressive ART; FAM-HIC, HIV-negative first-degree relatives of HIC; FAM-non-HIC, HIV-negative first-degree relatives of non-HIC; cART, Anti-Retroviral Therapy; NA, not applicable.

a Mean (SD) for Age; Median (IQR) for skewed continuous; n (%) for categorical.

b Wilcoxon rank-sum test for continuous; Chi-square test for categorical variables (Fisher's exact test was used when expected counts were <5).

c Unmeasurable, unquantifiable or <40 copies/mL.

d >40 copies/mL, exact quantification.

**Table 2:** Baseline characteristics of the 2000HIV-TRAINED substudy.

Variable	HIC^a^ n = 31	Non-HIC^a^ n = 30	p-value^b^	FAM-HIC^a^ n = 23	FAM-non-HIC^a^ n = 21	p-value^b^
**Sex**			0·40			1
Female	11 (35·5%)	7 (23·3%)		15 (65·2%)	14 (66·7%)	
Male	20 (64·5%)	23 (76·7%)		8 (34·8%)	7 (33·3%)	
**Age in years**	51·3 (14·7)	53·8 (10·6)	0·35	53·0 (13·3)	54·1 (19·0)	0·68
**Covid19 vaccination**			0·75			0·29
No	5 (16·1%)	6 (20·0%)		7 (30·4%)	3 (14·3%)	
Yes	26 (83·9%)	24 (80·0%)		16 (69·6%)	18 (85·7%)	
**Ethnicity**			0·93			0·80
Asian	2 (6·5%)	1 (3·3%)		0	0	
Black	4 (12·9%)	2 (6·7%)		2 (8·7%)	0 (0·0%)	
Hispanic	1 (3·2%)	1 (3·3%)		2 (8·7%)	1 (4·8%)	
Other	2 (6·5%)	2 (6·7%)		1 (4·3%)	1 (4·8%)	
White	22 (71·0%)	24 (80·0%)		18 (78·3%)	19 (90·5%)	
**CD4 latest n ×10^9^ cells/L**	0·76 (0·6, 1·08)	0·74 (0·56, 0·9)	0·32	NA	NA	NA
**CD4 Nadir n ×10^9^ cells/L**	0·52 (0·41, 0·66)	0·24 (0·09, 0·32)	2·3 × 10^−4^	NA	NA	NA
**VL latest**			0·35			NA
Undetectable^c^	27 (87·1%)	29 (96·7%)		NA	NA	
Detectable^d^	4 (12·9%)	1 (3·3%)		NA	NA	
**VL latest in copies/mL (for detectable means)**	99 (37·5, 208·5)	104 (104, 104)	1	NA	NA	NA
**HIV duration, years**	15 (11, 18·6)	14·3 (10·6, 20·9)	0·96	NA	NA	NA
**ART usage**			1·3 × 10^−5^			
Yes	12 (38·7%)	30 (100·0%)		NA	NA	
No	19 (61·3%)	0 (0·0%)		NA	NA	
**CMV serostatus**			1·00			0·76
Negative	2 (6·5%)	1 (3·3%)		10 (43·5%)	11 (52·4%)	
Positive	29 (93·5%)	29 (96·7%)		13 (56·5%)	10 (47·6%)	

HIC, HIV controllers; Non-HIC, normal progressors PLHIV on suppressive ART; FAM-HIC, HIV-negative first-degree relatives of HIC; FAM-non-HIC, HIV-negative first-degree relatives of non-HIC; cART, Anti-Retroviral Therapy; NA, not applicable.

a Mean (SD) for Age and HIV Duration; Median (IQR) for skewed continuous; n (%) for categorical.

b Wilcoxon rank-sum test for continuous; Chi-square test for categorical variables (Fisher's exact test was used when expected counts were <5).

c Unmeasurable, unquantifiable or <40 copies/mL.

d >40 copies/mL, exact quantification.

### Role of funders

The authors are part of the 2000HIV study, which is supported by ViiV Healthcare. ViiV Healthcare did not have any role in data quality control, statistical analyses, writing, or final interpretation of the data, but retained the right to review the content. No medical writer was employed in the preparation of this manuscript.

## Results

### HIV infection- and controller status affect the phenotype of circulating NK cells

**Fig. 1:**
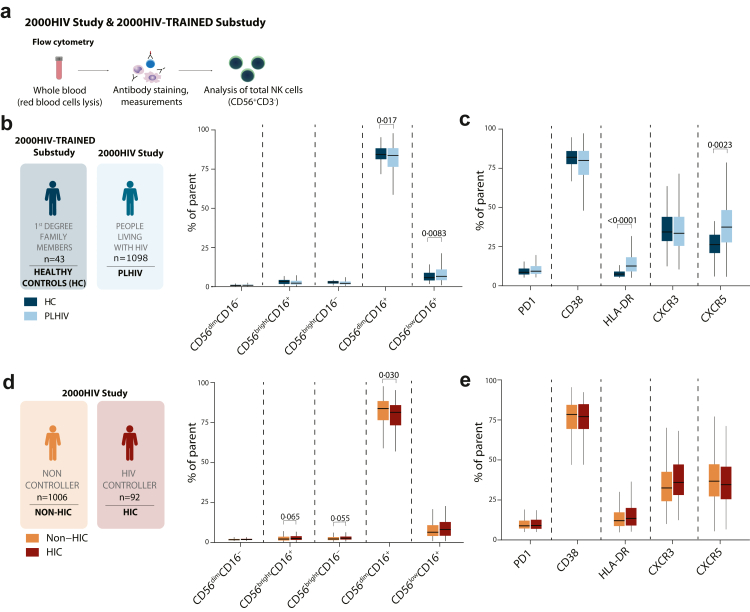
**Frequency and receptor expression of NK cell subpopulations are influenced by HIV infection and differ in individuals who can spontaneously control HIV. (a)** Workflow of whole-blood NK cell characterisation performed in the people living with HIV (PLHIV) from the 2000HIV-Discovery cohort and family members from the 2000HIV-TRAINED Substudy as a group of healthy controls (HC). **(b)** Percentages CD56^bright^CD16^−^, CD56^bright^CD16^+^, CD56^dim^CD16^+^, CD56^dim^CD16^−^, and CD56^low^CD16^+^ NK cell subpopulations assessed in whole blood of PLHIV (n = 1098) of the 2000HIV study and HC (n = 43) of the 2000HIV-TRAINED Substudy. **(c)** Percentages of CD45^+^CD3^−^CD56^+^ live cells expressing exhaustion markers PD1, CD38, the activation marker HLA-DR, and chemokine receptors CXCR5 and CXCR3 in whole blood of PLHIV and HC. **(d)** Percentages of CD56^bright^CD16^−^, CD56^bright^CD16^+^, CD56^dim^CD16^+^, CD56^dim^CD16^−^, and CD56^low^CD16^+^ NK cell subpopulations assessed in whole blood of HIV controllers (HIC) (n = 92) and non-controllers (non-HIC) (n = 1006) of the 2000HIV-Study. **(e)** Percentages of CD45^+^CD3^−^CD56^+^ live cells expressing exhaustion markers PD1, CD38, the activation marker HLA-DR, and chemokine receptors CXCR5 and CXCR3 in whole blood of HIC and non-HIC. Statistical analysis was performed using a linear regression model, corrected for sex, age, season of inclusion, time to lab, and COVID-19 vaccination. Data represented as median with interquartile range. Whiskers extend to 1·5 × IQR. Exact p-values are shown.

We first aimed to understand HIV-mediated NK cell alterations by assessing the distribution of the five main subpopulations of circulating NK cells based on the CD56 and CD16 expression in PLHIV (n = 1098) and healthy controls (n = 43) (Fig. 1b), with their characteristics described in Table 1. PLHIV consisted of virally suppressed individuals enrolled in the 2000HIV study. Healthy controls were enrolled for the 2000HIV-TRAINED substudy and consisted of 1st degree family members of PLHIV. Both cohorts were sampled, processed, and analysed in the same manner, enabling comparability of results. To understand overall activation or exhaustion status as well as homing capabilities, we also measured the expression of CD38, HLA-DR, PD-1, CXCR3, and CXCR5 on CD3-CD56^+^ NK cells (for gating strategy, see Figs. S2A and S3). We observed that the percentages of CD56^dim^CD16^+^ NK cells were lower (p = 0·017, β = −0·39), while CD56^low^CD16^+^ NK cells were higher in PLHIV than healthy controls (p = 0·0083, β = 0·43) (Fig. 1b). In addition, compared to healthy controls, circulating NK cells in PLHIV were predominantly activated (CD3^−^CD56^+^ HLA-DR^+^) (p < 0·001, β = 1·12) and had a more mature phenotype (CD56^low^CD16^+^) (Fig. 1c).^48^ Increased CXCR5^+^ NK cells (p = 0·0023, β = 0·47) were also observed in PLHIV (Fig. 1c), illustrating higher homing capabilities to secondary lymphoid organs.^49^

We subsequently sought to investigate whether these same NK cell features differed between HIC (n = 92) and non-HIC (n = 1006). HIC had lower frequencies of CD56^dim^CD16^+^ NK cells (p = 0·030, β = −0·24) cells and a trend towards higher percentages of CD56^bright^CD16^−^ and CD56^bright^CD16^+^ NK cells compared to non-HIC (p = 0·055, β = 0·19 and p = 0·065, β = 0·17, respectively) (Fig. 1d). While there were no differences in the expression of activation, exhaustion, or chemokine receptors between the groups (Fig. 1e), the increase in CD56^bright^ populations and the decrease in CD56^dim^CD16^+^ population in HIC suggest a shift in NK cell differentiation, with early-stage NK cells being characteristic of controller status.

### SNPs associated with HIV control downmodulate the expression of KIR2DL2/3 and its ligand HLA-C

**Fig. 2:**
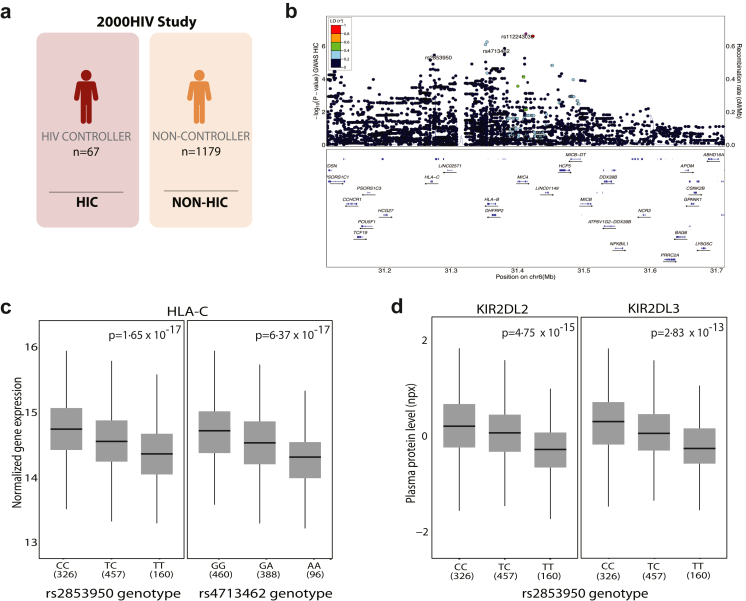
**SNPs in the MHC locus that are associated with HIV control downmodulate the gene expression and plasma levels of KIR2DL2/3 and its ligand HLA-C. (a)** A genome-wide association study (GWAS) was performed on 67 HIC and 1179 non-HIC from the 2000HIV study. **(b)** Regional association plot of SNPs in the MHC locus of chromosome 6 that are significantly associated with HIV control. **(c)** eQTL analysis based on the identified top SNPs rs2853950 and rs4713462 regulating the gene expression of the KIR2DL2/3 and its ligand HLA-C. **(d)** pQTL analysis of the top SNP rs2853950 regulating the protein levels of KIR2DL2 and KIR2DL3. Data represented as median with interquartile range. Whiskers extend to 1·5 × IQR. Exact p-values are shown.

The interaction of MHC class I molecules with NK cell receptors is well established, and this interaction is crucial for determining NK cell functionality.^50^ Moreover, previous genome-wide association studies have associated the presence of single nucleotide polymorphisms (SNPs) in the MHC class I region with spontaneous HIV control.^51^ Within the MHC locus, we identified three independent suggestive (p < 1 × 10^−5^, r^2^ < 0·1, kb < 500) lead SNPs associated with HIV control (n = 67 HIC, n = 1179 non-HIC) (Fig. 2a and b). Two approaches were used to gain further insights into the mechanisms related to the regulation of NK cell functionality. First, we used the summary statistics of an expression quantitative trait locus (eQTL) study in PBMCs from our cohort to pinpoint effects on gene expression. Among the three lead SNPs which were associated with HIV control in our cohort, we found that the protective allele of two of the SNPs, rs2853950-T (p_GWAS_ = 7·22 × 10^−6^; OR = 2·32) and rs4713462-A (p_GWAS_ = 1·37 × 10^−6^; OR = 2·41) were associated with decreased *HLA-C* expression in PBMCs from the individuals of our cohort (p_eQTL_ = 1·65 × 10^−17^, β_eQTL_ = −0·36 and p_eQTL_ = 6·37 × 10^−17^, β_eQTL_ = −0·37, respectively) (Tables 3 and 4, Fig. 2c). In addition, the SNP rs112243036 (p_GWAS_ = 1·82 × 10^−7^; OR = 3·28) was found to affect the expression of *MICA*, a ligand for the NK cell receptor NKG2D (p_eQTL_ = 1·47 × 10^−15^, β_eQTL_ = −0·54) (Tables 3 and 4, Fig. S11). Secondly, we made use of the plasma protein QTL (pQTL) summary statistics from our cohort to identify potential effects on circulating plasma protein levels. We identified that the SNP rs2853950-T, which decreases *HLA-C* gene expression, also downregulates KIR2DL2 (p_pQTL_ = 4·75 × 10^−15^, β_eQTL_ = −0·33) and KIR2DL3 (p_pQTL_ = 2·83 × 10^−13^, β_eQTL_ = −0·3) plasma protein abundance (Tables 3 and 5, Fig. 2d). Overall, these results indicate that HIV control protective alleles may modulate the NK cell receptor repertoire, including the expression of KIR2DL2/3.

**Table 3:** Single nucleotide polymorphisms (SNPs) associated with HIV control in Europeans of the 2000HIV cohort and expression and protein quantitative trait locus (eQTL, pQTL) of PBMCs and plasma proteomics, respectively.

SNP	Chr^a^	Position	p-value^b^	OR^c^	95% CI	Tested allele^d^	Alternative allele^e^	eQTL^h^^,^^i^^,^^l^; pQTL^j^^,^^k^
rs2853950	6	31268398	7·22 × 10^−6^	2·321	1·607–3·353	T	C	*HLA-C*^f^^,^^h^ (−), *HLA-DQA2*^g^^,^^h^ (+), *HLA-DRB6*^g^^,^^h^ (+), *HLA-G*^g^^,^^h^ (+), *HLA-H*^g^^,^^h^ (+), *HLA-L*^g^^,^^h^ (+), *HLA-A*^g^^,^^j^ (−), *KIR2DL2*^g^^,^^j^ (−), *KIR2DL3*^g^^,^^j^ (−), *LINC00243*^g^^,^^h^ (−)
rs4713462	6	31380039	1·37 × 10^−6^	2·412	1·687–3·447	A	G	*CCHCR1*^f^^,^^h^ (−), *HLA-C*^f^^,^^h^ (−), *HLA-G*^g^^,^^h^ (+), *HLA-H*^g^^,^^h^ (+), *MICB*^f^^,^^j^ (−), *VARS2*^g^^,^^h^ (+)
rs112243036	6	31412257	1·82 × 10^−6^	3·280	2·099–5·125	A	G	ENSG00000272221^f^^,^^h^ (−), *HLA-H*^g^^,^^h^ (+), *LRPAP1*^g^^,^^j^ (+), *MICA*^f^^,^^h^ (−), *MICB*^f^^,^^h^^,^^i^ (−)

a Chr, chromosome.

b p-value for association to HIV control, as determined by a logistic regression model adjusted for age, sex and the first five genetic PCs as covariates.

c OR, odds ratio.

d Reference allele associated with HIV control.

e Alternative allele.

f Cis gene (250 kb).

g Trans gene.

h Expression quantitative trait locus (QTL) results in PBMCs show a correlation between the lead SNP and the expression of the mentioned gene.

i Expression QTL results in PBMCs show a correlation between the proxy of the lead SNP (r^2^ > 0·8) and the expression of the mentioned gene.

j Protein QTL results in plasma show a correlation between the lead SNP and the expression of the mentioned protein.

k Protein QTL results in plasma show a correlation between a proxy of the lead SNP (r^2^ > 0·8) and the expression of the mentioned protein. Directions (+[increased expression]) (−[decreased expression]) of the QTLs are given with respect to the allele associated with HIV control.

l (+) increased expression; (−) decreased expression.

**Table 4:** eQTL summary statistics for lead HIV control SNPs in the MHC locus from both discovery and validation cohort (2000HIV study).

SNP	Beta value discovery	95% CI discovery	p-value discovery	Beta value validation	95% CI validation	p-value validation	*Gene name*
rs112243036	0·72	0·5885–0·8515	1·62 × 10^−25^	0·76	0·4866–1·0334	1·06 × 10^−7^	*HLA-H*
rs112243036	−0·94	−1·0654 to −0·8146	1·93 × 10^−44^	−1·04	−1·2958 to −0·7842	2·95 × 10^−14^	ENSG00000272221
rs112243036	−0·54	−0·6713 to −0·4087	1·47 × 10^−15^	−0·53	−0·8034 to −0·2566	2·07 × 10^−4^	*MICA*
rs4713462	0·49	0·4038–0·5762	9·55 × 10^−28^	0·56	0·3760–0·7440	1·00 × 10^−8^	*HLA-G*
rs4713462	0·26	0·1708–0·3492	1·29 × 10^−8^	0·23	0·0340–0·4260	2·09 × 10^−2^	*HLA-H*
rs4713462	0·27	0·1812–0·3588	5·87 × 10^−9^	0·19	0·0008–0·3792	4·69 × 10^−2^	*VARS2*
rs4713462	−0·27	−0·3504 to −0·1896	3·18 × 10^−11^	−0·26	−0·4286 to −0·0914	3·38 × 10^−3^	*CCHCR1*
rs4713462	−0·37	−0·4562 to −0·2838	6·37 × 10^−17^	−0·27	−0·4570 to −0·0830	4·96 × 10^−3^	*HLA-C*
rs2853950	0·38	0·2977–0·4623	2·86 × 10^−18^	0·31	0·1357–0·4843	4·94 × 10^−4^	*HLA-G*
rs2853950	0·5	0·4196–0·5804	2·22 × 10^−32^	0·54	0·3739–0·7061	7·90 × 10^−10^	*HLA-H*
rs2853950	0·46	0·3796–0·5404	1·57 × 10^−27^	0·57	0·4065–0·7335	8·69 × 10^−11^	*HLA-L*
rs2853950	−0·23	−0·3044 to −0·1556	3·19 × 10^−9^	−0·2	−0·3431 to −0·0569	7·60 × 10^−3^	*LINC00243*
rs2853950	−0·36	−0·4400 to −0·2800	1·65 × 10^−17^	−0·27	−0·4400 to −0·1000	2·34 × 10^−3^	*HLA-C*
rs2853950	0·25	0·1657–0·3343	1·17 × 10^−8^	0·45	0·2799–0·6201	5·11 × 10^−7^	*HLA-DRB6*
rs2853950	0·24	0·1577–0·3223	1·70 × 10^−8^	0·39	0·2239–0·5561	7·19 × 10^−6^	*HLA-DQA2*

eQTL was mapped using the inverse-rank transformed gene expression values using a linear model that included age, sex, BMI, seasonality, inclusion period, COVID vaccination, and centre of inclusion.

**Table 5:** pQTL summary statistics from for lead HIV control SNPs in the MHC locus both discovery and validation cohort (2000HIV study).

SNP	Beta value discovery	95% CI discovery	p-value discovery	Beta value validation	95% CI beta validation	p-value validation	Protein name
rs112243036	−0·87	−0·9944 to −0·7456	4·20 × 10^−38^	−0·82	−1·0758 to −0·5642	4·44 × 10^−10^	MICA/B
rs112243036	0·45	0·3240–0·5760	5·39 × 10^−12^	0·36	0·1042–0·6158	6·53 × 10^−3^	LRPAP1
rs4713462	−0·35	−0·4362 to −0·2638	3·75 × 10^−15^	−0·22	−0·3993 to −0·0407	1·50 × 10^−2^	MICA/B
rs2853950	−0·3	−0·3784 to −0·2216	2·83 × 10^−13^	−0·46	−0·6195 to −0·3005	2·99 × 10^−8^	KIR2DL3
rs2853950	−0·33	−0·4123 to −0·2477	8·07 × 10^−15^	−0·32	−0·4846 to −0·1554	2·05 × 10^−4^	HLA-A
rs2853950	−0·33	−0·4133 to −0·2467	4·75 × 10^−15^	−0·35	−0·5172 to −0·1828	6·57 × 10^−5^	KIR2DL2

pQTL was mapped using the inverse-rank transformed gene expression values using a linear model that included age, sex, BMI, seasonality, inclusion period, COVID vaccination, and centre of inclusion.

### HIV control is associated with increased CD56^bright^ NK cells expressing cytotoxicity receptors

The results of the whole blood flowcytometry and the GWAS data suggest that NK cell subpopulations and the NK cell receptor repertoire differ in HIC compared to non-HIC. To gain further insight into NK cell receptor surface expression on NK cell subpopulations, we performed an in-depth characterisation of CD56+ sorted NK cells in a selection of participants.

**Fig. 3:**
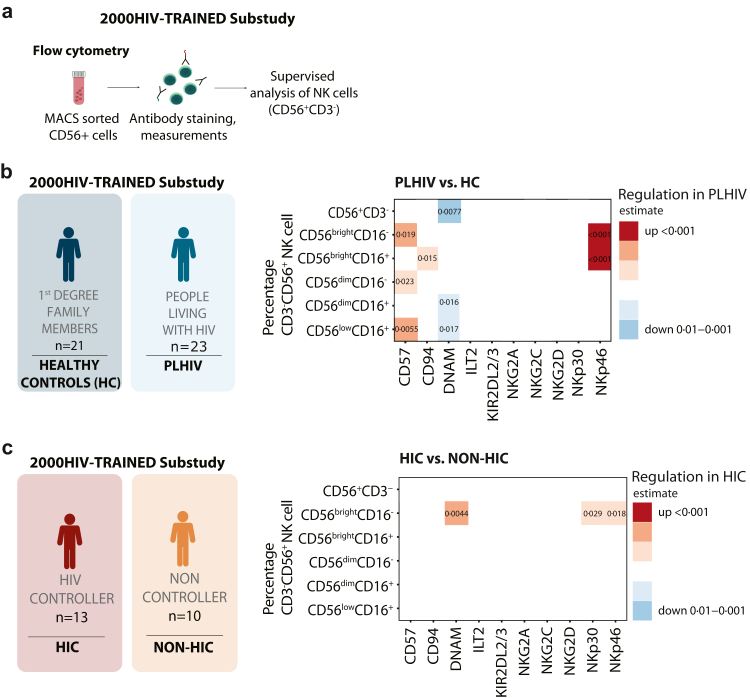
**NK cells of non-controllers on ART exhibit increased maturation towards CD56dim expressing CD57 compared to healthy controls, while HIC have increased cytotoxicity receptors on CD56bright NK cells. (a)** CD56^+^ NK cells were obtained through MACS isolation from PLHIV (n = 23), including both HIC (n = 13) and non-HIC (n = 10), and from HC (n = 21) part of 2000HIV-TRAINED Substudy. Flowcytometry was performed to determine the expression of CD57, CD94, DNAM, ILT2, KIR2DL2/3, NKG2A, NKG2C, NKG2D, NKp30, and NKp40 on CD3^−^CD56^+^NK cells. **(b)** Heatmap representing percentage of CD56^bright^CD16^−^, CD56^bright^CD16^+^, CD56^dim^CD16^+^, CD56^dim^CD16^−^, and CD56^low^CD16^+^ NK cells subpopulations expressing CD57, CD94, DNAM, ILT2, KIR2DL2/3, NKG2A, NKG2C, NKG2D, NKp30, and NKp40 between PLHIV and HC **(c)** and HIC vs. non-HIC. Statistical analysis was performed using a linear regression model, corrected for sex and age. Data represented as estimates. Exact p-values are shown.

We phenotyped CD56^+^ sorted NK cells in the individuals from the 2000HIV-TRAINED substudy, which included a selection of HIC (n = 13), non-HIC (n = 10) and healthy controls (n = 21) (Table 2). We evaluated the receptor expression associated with cytotoxicity (NKp46, NKp30), activation, and cytokine production (DNAM-1), stress response (NKG2D), memory (CD57, NKG2C, and CD94), and inhibitory function (NKG2A, ILT2, and KIR2DL2/3) among NK cell subpopulations (Fig. 3a, for gating see Figs. S4–S6A).^15^, ^16^, ^17^^,^^52^

We also assessed differences in subpopulations of circulating NK cells based on the CD56 and CD16 expression. Although no significant differences were observed between PLHIV and healthy controls (Fig. S6B), the same tendencies of having increased CD56^bright^ (p = 0·088) and decreased CD56^dim^CD16^+^ (p = 0·07) in HIC compared to non-HIC were observed (Fig. S6C). Compared to healthy controls, total CD56^+^CD3^−^ NK cells from PLHIV had lower DNAM-1 expression (p = 0·0077, β = 0·87). CD56^bright^CD16^−^ and CD56^bright^CD16^+^ NK cells from PLHIV showed higher expression of NKp46 (p = 0·00018, β = 1·17 and p = 0·00019, β = 1·15, respectively). CD56^bright^CD16^−^ (p = 0·019, β = 0·76), CD56^dim^CD16^−^ (p = 0·023, β = 0·69), and CD56^low^CD16^+^ (p = 0·0055, β = −0·65) NK cells exhibited higher CD57 expression than healthy controls. CD56^low^CD16^+^ NK cells from PLHIV had higher CD57 (p = 0·0055, β = 0·85) and lower DNAM-1 (p = 0·017, β = −0·76) expression than healthy controls (Fig. 3b). These changes in NK cell receptor expression capture the presence of cells with distinct functions, with PLHIV having CD56^bright^ NK cells with increased cytotoxicity capabilities and CD56^dim^CD16^+^ cells shifting towards a terminally differentiated phenotype (CD56^dim^CD16^+^CD57^+^).^53^

Comparing HIC and non-HIC, we observed increased frequencies of CD56^bright^CD16^−^ expressing DNAM-1 (p = 0·0044, β = 1·17), NKp30 (p = 0·029, β = 0·98), and NKp46 (p = 0·018, β = 0·84) in HIC. In contrast, no significant differences were found in the expression of the other functional receptors (Fig. 3c). Altogether, our results demonstrate that the phenotype of CD56^bright^ NK cells identified in HIV controllers show increased expression of cytotoxicity-associated functional receptors, while the NK cell compartment of non-controllers was characterised by terminally differentiated NK cells with both activating and inhibitory functions.

### Unsupervised clustering identified an increased expansion of NKG2C^+^ NK cells in PLHIV

**Fig. 4:**
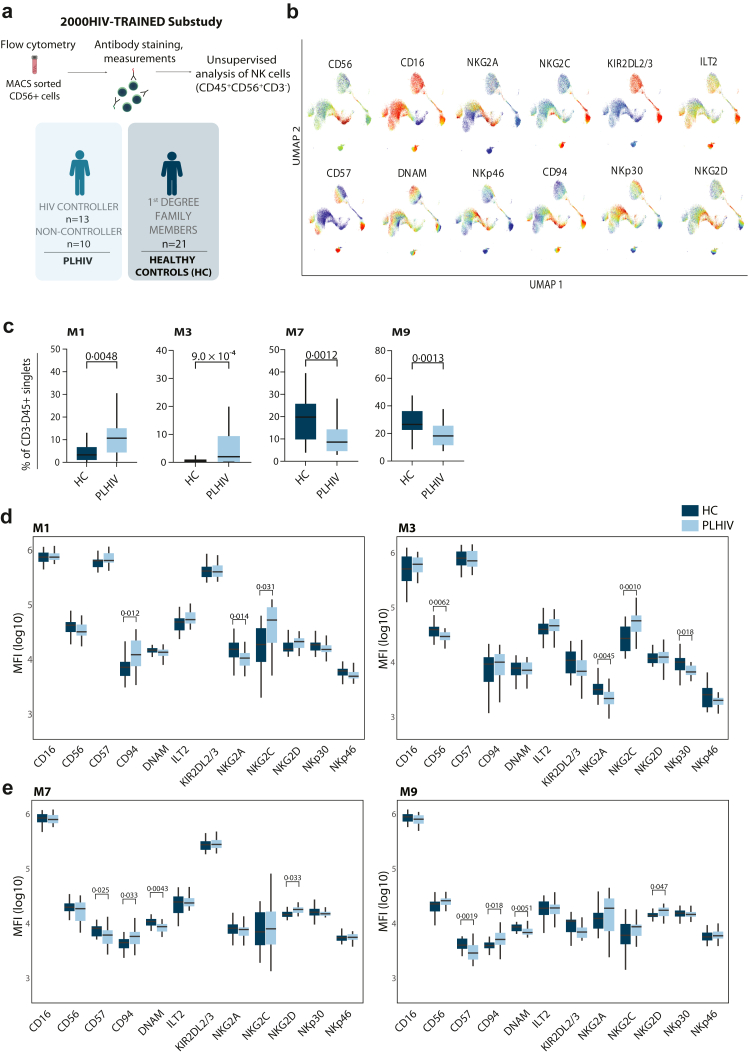
**Unsupervised analysis of NK cells with FlowSOM allows identification of differential metaclusters in PLHIV and healthy controls. (a)** CD56^+^ NK cells were obtained through MACS isolation from PLHIV (n = 23), including both HIC (n = 13) and non-HIC (n = 10) and HC (n = 21) part of the 2000HIV-TRAINED substudy. Flow cytometry was performed to determine the expression of CD57, CD94, DNAM, ILT2, KIR2DL2/3, NKG2A, NKG2C, NKG2D, NKp30, and NKp40 on CD3^−^CD56^+^NK cells and unsupervised analysis with dimension reduction using UMAP and FlowSOM metaclustering was employed. **(b)** Representation of marker expression within NK cells on a UMAP scale (representative data of one individual with HIV). **(c)** Comparison of M1, M3 percentages relative to total NK cells (CD45^+^CD3^−^CD56^+^) between HC and PLHIV. **(d)** Expression of CD16, CD56, CD57, CD94, DNAM, ILT2, KIR2DL2/3, NKG2A, NKG2C, NKG2D, NKp30, and NKp40 in M1 and M3 as well as **(e)** M7 and M9 among HC or PLHIV. MFI depicted as log(10) values. Data are represented as median with interquartile range. Whiskers extend to 1·5 × IQR. Significance was tested using a Wilcoxon rank-sum test. Exact p-values are shown.

We performed unsupervised flow cytometry analysis on CD56^+^ sorted NK cells from PLHIV and healthy controls (Fig. 4a) to elucidate the relationships among different NK cell subpopulations expressing multiple receptors. Using uniform manifold approximation and projection for dimension reduction (UMAP), we observed intricate cellular heterogeneity patterns based on the expression profile of the different functional markers (Fig. 4b). Based on receptor expression patterns, NK cell clusters were quantified by a self-organising map algorithm called FlowSOM. We identified 15 NK cell metaclusters (M) in our dataset (Fig. S7A), each with a unique combination of functional NK cell receptors. To identify NK cells characteristic of PLHIV, we quantified the percentages of CD56^+^ NK cells in each metacluster between PLHIV and healthy individuals. Increased percentages of NK cells in M1 (p = 0·0048) and M3 (p = 0·0009) were observed in PLHIV, while the percentages in M7 (p = 0·0012) and M9 (p = 0·0013) were decreased compared to healthy controls (Fig. 4c). Although no significant differences between PLHIV and healthy controls were found in the remaining clusters, the expression levels of several receptors differed between the groups (Fig. S7B–D). M1 and M3, both increased in PLHIV, showed higher NKG2C (p = 0·031 and p = 0·0010, respectively) and lower NKG2A (p = 0·014 and p = 0·0045, respectively) expression compared to healthy controls, suggesting a subpopulation of NK cells known as memory-like NK cells. In addition, M1 displayed higher CD94 expression in PLHIV compared to HC (p = 0·012) (Fig. 4d). In M3, CD56 (p = 0·0062) and NKp30 (p = 0·018) expression was lower in PLHIV compared to healthy controls. In contrast to that, M7 and M9 showed lower CD57 (p = 0·025, 0·0019) and DNAM (p = 0·0043, p = 0·0051) expression and higher CD94 (p = 0·033, p = 0·018) and NKG2D (p = 0·033, 0·047) expression in PLHIV compared to healthy controls (Fig. 4e). Using the unsupervised FlowSOM algorithm, we identified specific NK cell subpopulations that are altered in chronic HIV infections, showing changes not only in their proportions but also in their functional receptor expression.

### HIV and CMV co-infection induce distinct subsets of NKG2C+ NK cells, with differential expression of KIR2DL2/3

One of the hallmarks of memory NK cells commonly induced during viral infections, such as CMV, is the activating receptor NKG2C, identified as highly expressed in M1 and M3. CMV is a common co-infection in PLHIV, with seropositivity up to 95% in MSM-PLHIV.^27^^,^^29^^,^^30^^,^^32^ As CMV has a well-studied effect on NK cells, such as inducing the clonal expansion of memory-like NK cells, we aimed to distinguish the CMV-induced memory NK cells to assess the impact of chronic viral infections (CMV and HIV) on the NK cell repertoire.^27^ Of importance, all PLHIV included in our flow cytometry analysis (2000HIV-TRAINED study) are CMV seropositive (Table 2).

**Fig. 5:**
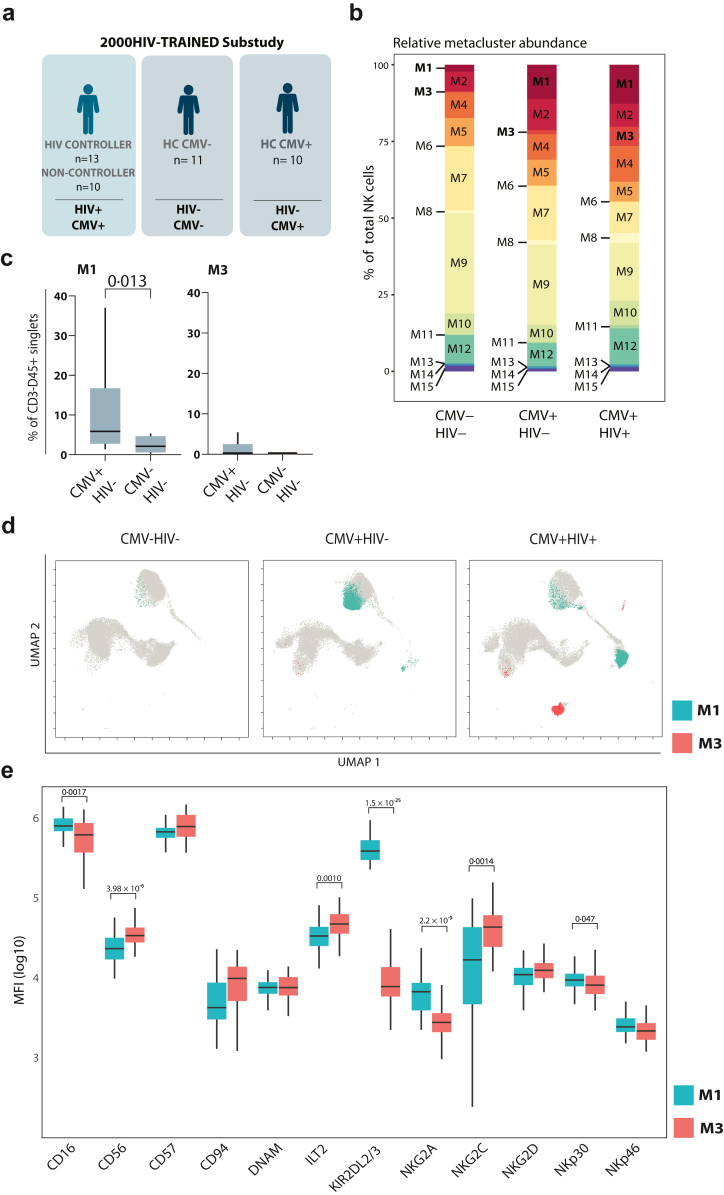
**CMV and HIV induce distinct memory NK cells with differential expression of KIR2DL2/3. (a)** Overview of PLHIV and HC of the 2000HIV-TRAINED substudy who are CMV-seropositive (n = 23 PLHIV, n = 10 HC) or -seronegative (n = 11 HC). **(b)** Average percentage of CD3^−^CD56^+^ NK cells within metaclusters among CMV−HIV−, CMV+HIV−, and CMV+HIV+ individuals. **(c)** Percentage of CD3^−^CD56^+^ NK cells within M1 and M3 in healthy controls stratified as CMV− and CMV+ individuals. **(d)** UMAP representation of M1 and M3 from a CMV−HIV−, a CMV+HIV−, and a CMV+HIV+ individual, respectively (representative data of one individual of each condition). **(e)** Expression of CD16, CD56, CD57, CD94, DNAM, ILT2, KIR2DL2/3, NKG2A, NKG2C, NKG2D, NKp30, and NKp40 among M1 vs. M3 of CMV+PLHIV. MFI is depicted as log(10) values. Data are represented as median with interquartile range. Whiskers extend to 1·5 × IQR. Significance was tested using a Wilcoxon rank-sum test. Exact p-values are shown.

To distinguish the effects of CMV and HIV in the subsets of memory NK cells, we stratified the HIV-negative, healthy control group by their CMV status (Fig. 5a). The visualisation of metacluster distributions across CMV−/HIV−, CMV+/HIV−, and CMV+/HIV+ groups revealed that M1 was more prevalent in CMV+/HIV− than in CMV−/HIV− individuals, whereas M3 increased only in CMV+/HIV+ individuals (Fig. 5b). Direct comparisons of NK cell frequencies confirmed that M1 was significantly expanded in CMV-seropositive versus CMV-seronegative healthy controls (p = 0·013) (Fig. 5c). In contrast, M3 remained consistently low in healthy controls, regardless of CMV status, and was significantly increased only in PLHIV. These findings were further supported by UMAP visualisation, which demonstrated a distinct expansion of M3 exclusively in CMV+/HIV+ individuals, with no comparable increase in CMV+/HIV− individuals (Fig. 5d). Our data suggest the presence of distinct memory subsets of NK cells. M1 resembles a subset of memory NK cells induced during CMV infection, whereas M3 is increased only in CMV+/HIV+ individuals. The observed increase in M3 may reflect an increase of a subpopulation that is specific to a co-infection of both CMV and HIV. The persistence of this subset in PLHIV with long-standing HIV infection suggests that it is not a transient population but rather a stable subset within the NK cell compartment. To understand the functional differences of M1 and M3, we assessed the individual expression of each functional receptor within memory NK cells from M1 and M3. The expression of CD16 (p = 0·0017) and NKp30 (p = 0·047) decreased in M3 compared to M1, while CD56 (p = 3·98 × 10^−6^) and ILT2 (p = 0·0010) increased. In addition, M3 had lower expression of the inhibitory NKG2A (p = 2·2 × 10^−9^) and KIR2DL2/3 (p = 1·5 × 10^−25^) receptors, whereas the NKG2C (p = 0·0014) expression was higher in comparison to M1 (Fig. 5e). Because KIR2DL2/3 expression was visually and statistically significantly reduced in M3, our results identify KIR2DL2/3 as the primary differentiating marker between CMV- and HIV-induced memory NK cells. Thus, we demonstrate that HIV-induced memory NK cells (M3) are characterised as NKG2A^low^NKG2C^high^ILT2^high^KIR2DL2/3^low^, while the CMV-induced memory NK cells (M1) express NKG2A^high^NKG2C^high^ILT2^high^KIR2DL2/3^high^.

Collectively, these findings suggest that while both CMV and HIV influence NK cell differentiation, M1 represents a classical CMV-driven memory-like NK cell subset, whereas M3 reflects a HIV-associated memory-like NK cell population that emerges in the setting of CMV and HIV co-infection.

### NKG2C^high^ILT2^high^KIR2DL2/3^low^ NK cells exhibit enhanced IFNγ production via epigenetic reprogramming in HIC

**Fig. 6:**
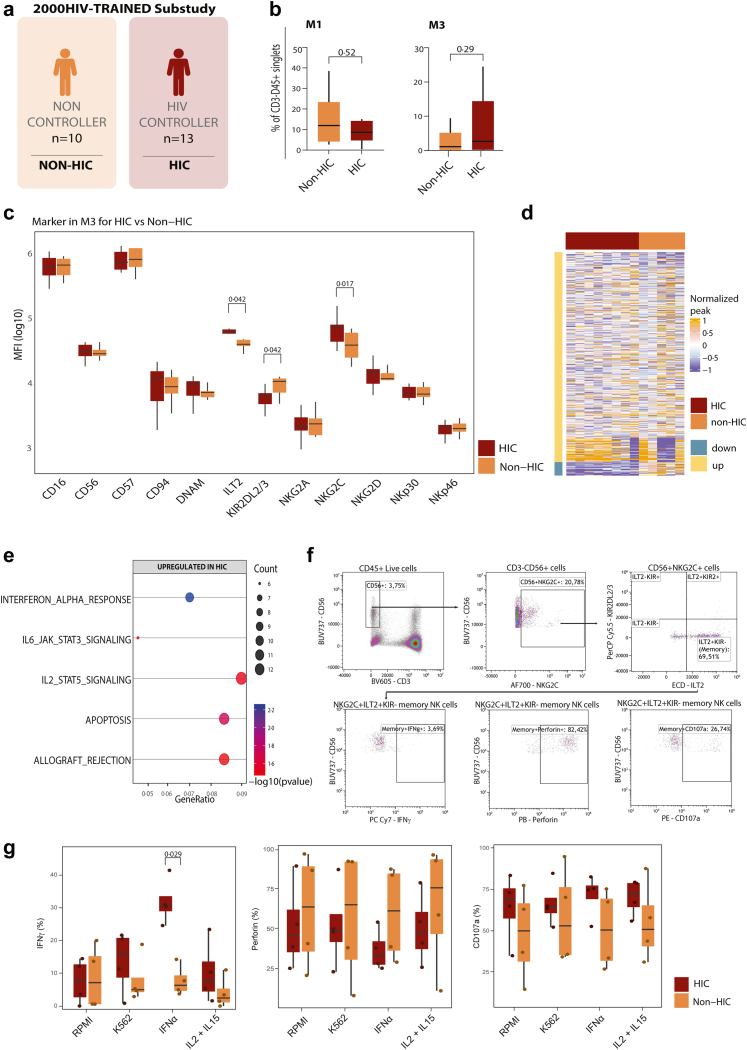
**NK cells from HIC show memory-like features with differential receptor expression of NKG2C, ILT2 and KIR2DL2/3 and are more responsive to IFNα stimulation. (a)** Overview of HIC and non-HIC from who flowcytometry data CD3^−^CD56^+^ NK cell data were available. **(b)** Frequencies of CD3^−^CD56^+^ NK cells between HIC (n = 13) and non-HIC (n = 10) within M1 and M3. Heatmap comparing the MFI expression of NK cell markers within M1, M3, and M8 in HIC and non-HIC. **(c)** Expression of CD16, CD56, CD57, CD94, DNAM, ILT2, KIR2DL2/3, NKG2A, NKG2C, NKG2D, NKp30, and NKp40 among HIC vs. non-HIC in M3. MFI depicted as log(10) values. Boxes represent interquartile ranges (IQR) with medians; whiskers show 1·5 × IQR. **(d)** Heatmap showing the normalised H3K4me3 deposition at promoters of CD3^−^CD56^+^ sorted NK cells from HIC (n = 8) and non-HIC (n = 5). **(e)** Functional enrichment of up- and down-regulated regions in NK cells from HIC compared to non-HIC using the Hallmark database. **(f)** PBMCs of 4 HIC and 4 non-HIC were activated for 10 h with IL-2 and IL-15 or left unstimulated (RPMI) and afterwards co-cultured with either MHC-deficient K562 cell lines for 4 h or activated with IFNα. Flowcytometry was performed afterwards for intracellular (IFNγ and Perforin) and extracellular (CD107a) functional markers. Memory NK cells were gated for NKG2C+ILT2+KIR2− NK cells. **(g)** Expression of functional markers IFNγ, perforin and CD107a in memory NK cells between HIC and non-HIC. Data are represented as median with interquartile range. Whiskers extend to 1·5 × IQR. Significance was tested using a Wilcoxon rank-sum test. Exact p-values are shown.

After identifying M1 as a consequence of CMV infection and M3 as a consequence of a co-infection of CMV and HIV, we aimed to evaluate if the frequency of these metaclusters differed between HIV controllers and non-controllers (Fig. 6a). No differences in frequencies of memory NK cell subsets (M1 and M3) were observed in HIC versus non-HIC (Fig. 6b), nor in any of the other NK cell metaclusters (Fig. S8A). However, when assessing the expression of functional receptors within the metaclusters between HIC and non-HIC, M2, M10, M11, and M12 displayed higher ILT2 (p = 0·036), CD56 (p = 0·012), NKp30 (p = 0·0046), and DNAM-1 (p = 0·049) expression in HIC, respectively (Fig. S8B). Within M3, previously characterised as HIV-induced, the expression of NKG2C (p = 0·017) and ILT2 (p = 0·042) was higher in HIC compared to non-HIC, while the KIR2DL2/3 (p = 0·042) expression was lower (Fig. 6c). These findings suggest that while the quantity of M3 does not differ between HIC and non-HIC, the individual expression of the functional receptors composing M3 reflects differences in NK cell regulation between these clinical phenotypes.

The mechanisms involved in NK cell memory rely not only on the expression of receptors but have also been attributed to changes in chromatin accessibility, facilitating rapid expansion and production of effector molecules.^27^^,^^31^ Memory functions in innate immune cells are frequently associated with epigenetic changes and modifications in chromatin accessibility.^54^^,^^55^ Therefore, we evaluated chromatin accessibility in sorted NK cells between HIC (n = 8) and non-HIC (n = 5) by measuring H3K4me3 levels in the promoter regions of immune-related genes. H3K4me3, a hallmark of innate immune memory, primes immune genes for transcription.^56^ CD56^+^ sorted NK cells from HIC showed increased accumulation of H3K4me3 at gene promoter regions compared to non-HIC, with 502 up- and 32 downregulated regions observed in HIC (Fig. 6d). The primed regions in HIC were enriched for genes of the “Hallmark Response to Interferon alpha” (p = 0·0054), “Hallmark IL-6 JAK STAT3 signalling” (p = 0·034), “Hallmark IL-2 JAK STAT5 signalling” (p = 0·030), “Hallmark Apoptosis” (p = 0·018) and “Hallmark Allograft rejection” (p = 0·033) pathways (Fig. 6e). Using other databases, pathways enriched in HIC included “MAPK signalling pathway”, “Efferocytosis,” and “Apoptosis” (KEGG database). The GO-BP database pathways included upregulation of “immune response-regulating cell surface receptor signalling pathway” and “adaptive immune response”, while “Regulation of growth”, “Histone modification” and “Cell growth” were downregulated (Fig. S9A).

To investigate if the observed changes in H3K4me3 accumulation was the result of increased production of NK cell stimulating factors, we measured IL-2, IL-15, and IL-18 secretion in PBMCs of HIC (n = 29), compared to non-HIC (n = 30) and healthy controls (n = 41), upon stimulation with (HIV-Env), CMV (CMV-pp65) or the TLR-7 agonist Imiquimod (IMQ) for 24 h. Although HIV-Env stimulation resulted in increased production of IL-2, IL-15, or IL-18 compared to the unstimulated RPMI condition, no differences were observed between HIC and non-HIC (Fig. S10A). Likewise, the exposure of PBMCs to CMV-pp65 and IMQ resulted in increased secretion of IL-15 and IL-18 in comparison to unstimulated controls, whereas no differences between HIC and non-HIC were observed (Fig. S10B and C). IFNα and IL-12 levels were below the detection limit of the assay. Our results indicate that PBMCs from HIC do not produce higher levels of NK cell-stimulating cytokines (IL-2, IL-15, and IL-18) compared to non-HIC.

We next hypothesised that NK cells from HIC are more responsive to NK cell-stimulating cytokines such as IFNα, IL-2, and IL-15 due to their differential H3K4me3 deposition in the “IL-2 JAK STAT5” and “Response to Interferon alpha” signalling pathways. Therefore, we assessed whether the changes in the profile of H3K4me3 of NK cells from HIV controllers would lead to increased IFNγ production and cytotoxicity as measured by CD107a and perforin release in the presence of IL-2/IL-15, IFNα, and K562 cells, a well-established model to induce NK cell activation via flow cytometry. To directly connect the NK cell functions to the chromatin accessibility data, we selected PBMCs of HIC (n = 4) and non-HIC (n = 4) that were previously characterised regarding their H3K4me3 profile. Exposure of NK cells to IL-2 and IL-15 led to a trend towards increased expression of intracellular IFNγ (p = 0·057) by total CD56^+^ NK cells in HIC compared to non-HIC, while no significant differences in CD107a or perforin expression were observed (Fig. S9D). To test whether the increased responsiveness to NK cell stimulation by NK cell-stimulating cytokines was specific to memory-NK cells, we compared effector molecule production of HIV-associated memory-like NK cells. For this purpose, we gated for intracellular effector molecule production in NKC2C+ILT2+KIR2DL2/3− NK cells (Fig. 6f). Memory-like NK cells from HIC produced more IFNγ (p = 0·029) than those of non-HIC upon stimulation with IFNα. While the production of perforin was overall lower in memory-like NK cells of HIC, albeit not significantly. These results show that memory-like NK cells from HIC respond with increased IFNγ production upon stimulation with IFNα, likely facilitated by the presence of H3K4me3. This suggests that epigenetic reprogramming enhances the antiviral responses of NK cells from HIC.

**Fig. 7:**
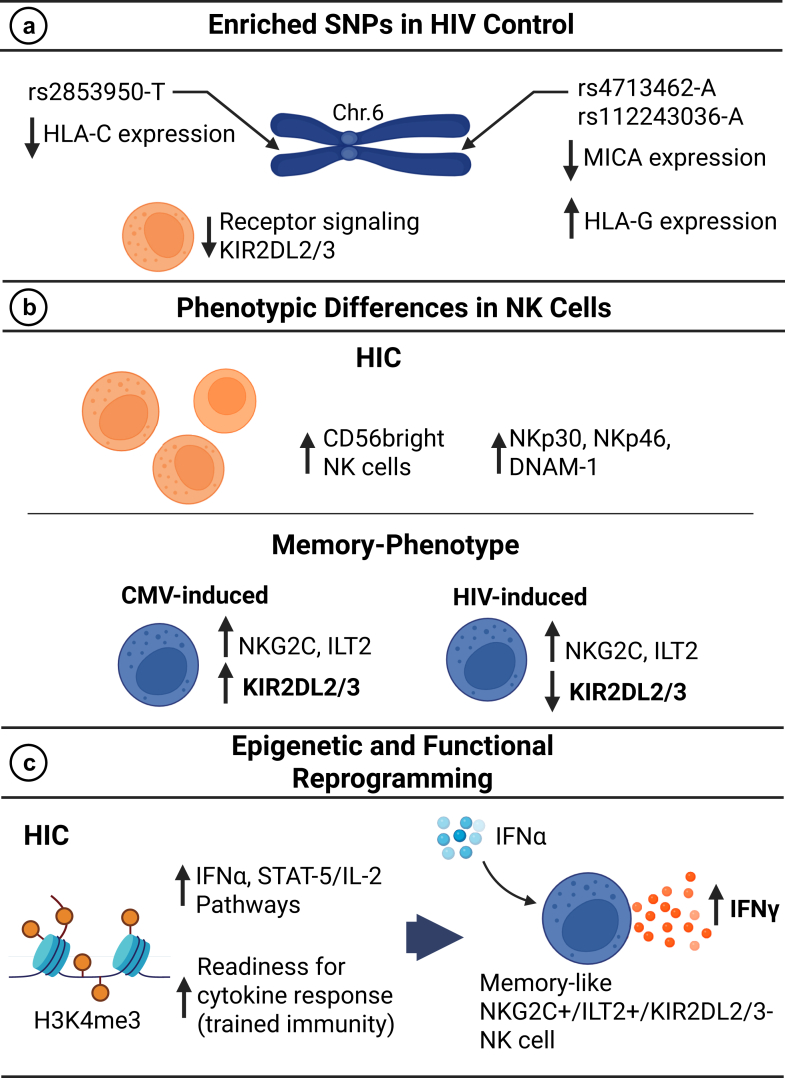
**Overview–distinct genetic, phenotypic, and functional features of NK cells in HIC. (a)** SNPs associated with HIV control are enriched near *HLA-C* and *MICA/HLA-G* loci on chromosome 6, with potential implications for reduced KIR2DL2/3 expression. **(b)** Phenotypic profiling of NK cells in HIC reveals an increase in Cd56bright cells and activating receptor expression (NKp30, NKp46, DNAM-1) (top). Memory-like NKG2C^+^ NK cells differ based on viral exposure: CMV-induced cells show increased KIR2DL2/3, whereas HIV-induced cells in HIC lack this upregulation (bottom). **(c)** NK cells in HIC exhibit epigenetic reprogramming (e.g., increased H3K4me3 at effector loci) and enhanced IFNα signalling, leading to a heightened IFNγ response upon stimulation, indicative of trained immunity. HIC, Spontaneous HIV-1 Controller; SNP, Single Nucleotide Polymorphism; CMV, Cytomegalovirus; IFN, Interferon; NKp30, Natural Cytotoxicity Receptor 3; NKp46, Natural Cytotoxicity Receptor 1; NKG2C, Natural Killer Group 2C receptor; ILT2, Immunoglobulin-like transcript 2; KIR2DL2/3, Killer-cell immunoglobulin-like receptor 2DL2/3; DNAM-1, DNAX accessory molecule-1; H3K4me3, Histone 3 lysine 4 trimethylation; STAT, Signal Transducer and Activator of Transcription. Figure created with Biorender.com.

Collectively, our data demonstrate distinct and coordinated adaptations in NK cells from HIV-1 controllers, which are summarised in Fig. 7. Our results demonstrate that NK cells of HIV controllers include a unique repertoire of functional memory NK cells characterised by changes in receptor expression (NKG2C^high^ILT2^high^KIRDL2/3^low^). In addition, we propose that the epigenetic programming results in increased response to NK cell-stimulating molecules, specifically IFNα. We propose that this phenotype of NK cells is essential for the spontaneous control of HIV replication.

## Discussion

Our study demonstrates that HIV infection impacts NK cell functionality differently in normal progressors on ART (non-controllers) compared to spontaneous HIV controllers. Compared to healthy individuals, NK cells from non-controllers presented features of a more mature NK subset, with a combination of receptors associated with activating and inhibitory functions. On the other hand, the maturation profile of NK cells from HIV controllers was comparable to that of healthy controls, but with increased expression of cytotoxicity receptors. Additionally, we identified a unique NK cell subpopulation (NKG2C^high^ ILT2^high^ KIR2DL2/3^low^) in PLHIV that was absent in healthy controls. While this subpopulation did not differ in frequency between HIV controllers and non-controllers, its receptor expression profile—characterised by higher levels of NKG2C and ILT2 and lower levels of KIR2DL2/3—was more pronounced in HIV controllers, suggesting distinct NK cell regulation in this group. In addition, we identified epigenetic modifications that were linked to increased responsiveness of these memory NK cells and increased production of IFNγ in spontaneous HIV controllers.

In our large cohort, the shift in NK cell subsets in PLHIV reflects not only statistical significance but also clinically meaningful differences. For example, PLHIV exhibited a 43% higher frequency of CD56^low^CD16^+^ NK cells compared to healthy controls (β = 0·43, 95% CI 0·20–0·66, p = 0·0083), indicating a pronounced skewing toward a more mature, potentially exhausted phenotype that may impair viral clearance. Conversely, HIV controllers showed a 24% reduction in CD56^dim^CD16^+^ NK cells relative to non-controllers (β = −0·24, 95% CI −0·45 to −0·03, p = 0·030), consistent with preservation of less differentiated NK subsets linked to durable viral suppression. At the genetic level, carriers of the rs2853950 protective allele had more than double the odds of spontaneous HIV control (OR = 2·32, 95% CI 1·70–3·18, p_GWAS = 7·22 × 10^−6^), a finding that is mechanistically supported by a corresponding decrease in HLA-C expression (β = −0·36, 95% CI −0·45 to −0·27, p_eQTL = 1·65 × 10^−17^). Together, these effect sizes and confidence intervals demonstrate that our observations go beyond nominal significance, revealing differences of a magnitude likely to impact patient outcomes and informing potential strategies to harness NK cells therapeutically.

The reduced expression of KIR2DL2/3, which distinguished these memory NK cells from CMV-induced NK cells, may be driven by HIV control protective alleles in the MHC locus. Additionally, we demonstrated that NK cells from HIV controllers were primed for transcription, as promoter regions of genes in the IFNα, IL-2, and IL-6 pathways showed increased H3K4me3 deposition, facilitating secondary responsiveness upon stimulation. This supports the presence of NK cells with memory features as one of the mechanisms contributing to the persistence of HIV control status.

In this study, we determined the impact of HIV infection on NK cell differentiation and functionality by assessing the phenotypical differences between PLHIV and uninfected healthy controls. In line with previous studies, our data emphasise HIV exposure as one of the factors associated with an altered composition of circulating NK cells.^11^^,^^48^^,^^57^ NK cells are often activated or present signs of exhaustion during HIV infection.^58^^,^^59^ We report an increase of NK cells expressing HLA-DR^+^ and CXCR5^+^ in PLHIV, which are subsets of NK cells known to be associated with chronic inflammation and migration to lymph nodes in response to simian immunodeficiency virus (SIV) infection, respectively.^60^^,^^61^ In addition, we identified signatures in NK cells of PLHIV indicating changes in maturation and functional capabilities. NK cells of PLHIV presented with characteristics of terminally differentiated cells, measured by the increased proportions of CD56^low^CD16^+^ expressing the maturation and memory marker CD57 and reduced expression of the cytotoxicity-associated DNAM-1 marker.^11^ These functional alterations lead to reduced NK cell-mediated killing mechanisms, thereby impairing the elimination of HIV-infected cells in PLHIV.^62^^,^^63^ Our findings differ from prior studies reporting expanded NKG2C+ NK cells in PLHIV in comparison to healthy controls.^64^^,^^65^ This difference likely arises from methodological differences: while earlier work focused on unadjusted comparisons, our analysis adjusted for sex and age, unmasking confounding by CMV seroprevalence. CMV is a potent driver of NKG2C+ expansions, and its higher prevalence in PLHIV (93·5% vs. 52·3% in controls) introduces bias when comparing cohorts.^33^ After adjustment, the dominant CMV effect likely obscured HIV-specific signals. However, our unsupervised analysis identified a distinct NKG2C+ILT2+CD57+ subset enriched in PLHIV, enabling more accurate identification of highly specialised NK cell subsets. In addition, a cross-sectional study has reported the expansion of CD56^dim^CD16^−^ NK cells lacking NKp30 and NKp46 expression in PLHIV receiving ART.^66^ In fact, contrary to normal progressors on ART, we demonstrated that NK cells from HIC were enriched with signatures of early-stage differentiated cells measured by increased proportions of CD56^bright^ NK cells expressing both NKp30 and NKp46-cytotoxicity-associated markers. Taken together, we propose that the persistence of a less differentiated population of CD56^bright^NK cells with cytotoxic properties may favour the spontaneous control of HIV replication. On the other hand, the absence or decreased exposure to HIV antigens during the chronic phase of the disease, which is a well-known characteristic of HIC, may contribute to the development of a distinct phenotype of NK cells in this group of PLHIV.^67^^,^^68^

Using unsupervised analysis, we identified the expansion of a unique subset of NK cells in PLHIV characterised by increased NKG2C, ILT2, and decreased KIR2DL2/3 expression. Increased NKG2C expression on NK cells of PLHIV driven by antigen exposure has been reported previously.^69^, ^70^, ^71^ In addition to increased NKG2C expression, the subset identified in our study also exhibited other key hallmarks of memory NK cells, including high CD57 expression, epigenetic modifications, enhanced effector functions, and stable persistence in PLHIV with chronic, long-term infection, further supporting its role as a memory NK cell subset.^27^^,^^33^^,^^72^^,^^73^ In addition, Jost et al. recently described the importance of the NKG2/HLA-E axis in memory responses in HIV infections, highlighting the role for NKG2C in memory NK cells.^36^

Our findings align with Hearps et al., demonstrating that adaptive NK cells persist for years in PLHIV despite suppressive therapy and expand rapidly during acute infection.^65^^,^^74^ The increase of NKG2C+ NK cells is often attributed to CMV co-infection due to the high frequency of CMV-seropositivity among PLHIV.^64^^,^^75^ However, the persistence of adaptive NK cells in early-treated PLHIV and our comparison with HIV−CMV+ individuals shows a distinct NK cell subpopulation in PLHIV, characterised by lower KIR2DL2/3 expression.^74^ Tomescu et al. previously demonstrated that CD57-positive NK cells from PLHIV, irrespective of NKG2C expression, exhibited significantly enhanced degranulation and IFN-γ production against heterologous gp120-coated ADCC targets coated with HIV reference plasma.^25^ While we did not evaluate ADCC responses towards HIV-specific targets, our data shows an increased IFNγ production of NKG2C+ NK cells in HIV controllers, suggesting a more effective anti-viral response.

Viral infections other than CMV and HIV have also been found to induce memory NK cells. An infection with SARS-CoV-2 was found to induce CD57+NKG2C+ NK cells, and NKG2C+ NK cells in humans infected with hantavirus showed memory-like characteristics.^29^^,^^76^ Collectively, these studies suggest that the expansion of memory NK cells, especially those expressing NKG2C, is an important mechanism against viral infections. The present study provided the opportunity for a comprehensive characterisation of NK cell receptor expression across various NK cell subsets between non-controllers and HIV controllers, as well as CMV seropositive and CMV seronegative individuals with or without HIV infection. With the current study design, we were able to identify the repertoire of NK cell receptor expression associated with CMV and HIV co-infections. However, as our cohort lacks an HIV+/CMV− group, we cannot conclusively isolate the specific contribution of HIV from the combined effects of HIV and CMV co-infection. Importantly, as also reported by others, most PLHIV are also co-infected with CMV, making the HIV+/CMV+ population of our study representative of the majority of PLHIV.^32^ Future studies prioritising the inclusion of HIV+/CMV− individuals are of interest to better delineate the specific impact of HIV on the NK cell repertoire. Importantly, we identified KIR2DL2/3 as a distinguishing marker between NK cells influenced by CMV in HIV−/CMV+ individuals and those shaped by the combined effects of HIV and CMV in co-infected PLHIV. Previous studies have reported increased expression of KIR2DL2/3 on NK cells during CMV infection, attributing KIR2DL2/3 expressing cells with a memory function.^77^ KIR2DL2/3 is an inhibitory receptor known to interact with the MHC-C molecule on HIV-infected cells.^78^ The engagement of the MHC-C-HIV-Gag complex with KIR2DL2 on NK cells leads to functional NK cell inhibition, therefore favouring the escape of virus-infected cells from NK cell-mediated lysis.^50^ In addition, this MHC-C-HIV-Gag complex stabilises the expression of HLA-C on the surface of infected cells, favouring NK cell inhibition mechanisms through HLA-C-KIR2DL2 interaction.^78^

Genetic variants in the MHC class I molecules have been associated with the phenotype of spontaneous controllers.^51^ Here, we provide evidence for the influence of these SNPs on the NK cell function. The rs2853950-T allele, identified as associated with HIV control, leads to the downmodulation of *HLA-C* gene expression in PBMCs. In addition, we demonstrated that this same rs2853950-T allele is a trans-QTL for the KIR2DL2/3 protein abundance in plasma. Thus, our study suggests a contribution of genetic variants in the MHC class I locus as one of the factors favouring the development of memory NK cells with reduced inhibitory receptor expression in HIV controllers. Moreover, the alleles associated with HIV control, rs2853950-T, rs4713462-A, and rs112243036-A, were also demonstrated to impact the expression of other well-known NK cell ligands, including *MICA/MICB* and *HLA-G*.^79^^,^^80^ While the rs2853950-T and rs4713462-A were associated with increased *HLA-G* expression in PBMCs, rs4713462-A led to decreased MICA/B plasma levels. Additionally, the rs112243036-A allele was associated with both decreased *MICA* gene and MICA/B protein expression. We propose two synergistic mechanisms underpinning this association: (1) Reduced HLA-C expression, driven by SNPs in HIV controllers (HIC), may lower inhibitory KIR2DL2/3 signalling by decreasing ligand availability, thereby disinhibiting NK cells and lowering activation thresholds.^81^ (2) Reduced MICA/B expression in HIC may limit proteolytic shedding, preserving membrane-bound ligands on infected cells and maintaining NKG2D-mediated recognition.^82^ While NKG2D surface expression was unchanged, enhanced ligand availability could sustain cytotoxic signalling, countering HIV immune evasion strategies.^83^ While our data suggest plausible mechanisms, further mechanistic studies are warranted.^82^ The current configuration of genetic variants in HIV controllers may influence the functional characteristics of NK cells that expand in response to viral encounters, affecting their response to other viral infections like CMV. While CMV infection may trigger the expansion of NK cells with inhibitory functions favouring its persistence and latency, this may not occur in HIV controllers, as our findings identify the presence of memory NK cell responses, which are less inhibitory than those observed in non-controllers.

NKG2C-expressing memory-like NK cells have been previously associated with HIV control, correlating with a low viral set point.^84^, ^85^, ^86^ HLA-E, a non-classical MHC class I molecule, plays a pivotal role in this interaction by presenting peptides to the activating receptor NKG2C, thereby modulating NK cell function.^85^^,^^86^ Recent work by Jost et al. (2023) highlights the critical role of the NKG2/HLA-E axis in enhancing NK cell cytotoxicity and antiviral activity in chronic viral infections, including HIV (Jost et al., 2023).^71^ From a functional perspective, memory NK cells, including those reported in CMV, cytokine-induced or related to pregnancies, are known to secrete higher amounts of IFN-γ.^27^^,^^31^ In line with previous studies, we provide evidence that NKG2C+ILT2+KIR2DL2/3− memory NK cells from HIV controllers are prone to increased IFNγ production upon stimulation, specifically with IFNα. Such an increase in IFNγ may be advantageous for HIV control, as its paracrine effects on other immune cells, including dendritic cells and monocytes/macrophages, trigger the induction of interferon-induced genes, which are well-known for their anti-viral properties.^87^^,^^88^

Importantly, our findings also argue for the presence of epigenetic memory in HIV controllers, possibly contributing to the persistence of long-lived NK cells that are readily able to respond with increased production of effector molecules. Innate immune cells exhibiting memory function have been termed *trained immunity*.^89^ We demonstrate that NK cells from HIV controllers are epigenetically primed for increased responses to stimulants acting via IFNα, IL-6, IL-2, and apoptosis pathways. The importance of IFNα in inducing a protective NK cell phenotype towards HIV control was previously reported in a study in which pegylated IFNα2a was administered in combination with ART, resulting in plasma HIV control and reduction of integrated HIV DNA.^90^ Our study shares key observations with the work of Sanchez-Gaona et al., who described a highly functional NK cell population in elite controllers marked by the co-expression of NKG2C and NKG2A.^86^ Their study underscores the enhanced antibody-dependent cellular cytotoxicity (ADCC) of these NK cells and their antiviral function. However, our study builds on this by offering insights into the epigenetic landscape of HIV-specific NK cells in controllers. Not only can we report a clear distinction between CMV-induced and HIV-induced memory NK cell populations, but we also show that H3K4me3 deposition is enhanced in genes associated with the IFNα, IL-15, and IL-2 pathways, which could help explain the enhanced antiviral functionality observed in NK cells from HIV controllers. This epigenetic priming suggests a deeper level of immune memory in NK cells from HIV controllers, potentially contributing to long-term HIV control. Such effects were further reported to be associated with increased NK cell activity and downmodulation of KIR2DL1 and KIR2DL2/3 expression, which was also associated with HIV control in our study and the differentiating receptor between CMV- and HIV-induced memory NK cells.^90^ Moreover, another recent study reported the presence of NK cells with elevated activating histone modifications at the IL-2/IL-15 receptor β chain and the *BCL2* gene loci in elite controllers. This led to increased NK cell cytotoxicity to paracrine IL-15 secretion, which coincided with higher IL-15 transcriptional levels by myeloid dendritic cells from spontaneous controllers^91^ IL-15, together with IL-2 and IL-18, are cytokines known to induce NK cell expansion; however, in the present study, the secretion of IL-15, IL-2, and IL-18 by PBMCs was comparable in controllers versus non-controllers upon stimulation with viral-mimicking ligands.^92^ While the heterogeneity in chromatin accessibility within the different NK cell subsets was not addressed in the present study, future studies employing single-cell epigenomics and longitudinal sampling are needed to clarify these relationships while minimising confounding from bulk analyses.

Despite the comprehensive nature of this study, several limitations must be considered. The absence of an HIV+/CMV− group limits the ability to isolate the specific effects of HIV infection from those of CMV co-infection, introducing potential confounding factors. Additionally, the cross-sectional design restricts our ability to infer causality or capture temporal changes in NK cell phenotype and function. While we observed phenotypic alterations in NK cells, the lack of functional assays, such as ADCC against HIV-specific targets, prevents direct validation of the observed changes in antiviral activity. Furthermore, bulk epigenetic analyses may obscure subset-specific variability, and single-cell approaches would provide more accurate insights. These limitations suggest that future studies incorporating longitudinal designs, functional assays, and single-cell analyses are necessary to further elucidate the role of NK cells in HIV control.

However, despite these limitations, this study represents significant value to the field by providing a detailed characterisation of NK cell subsets in the context of HIV and CMV co-infection and HIV control. We demonstrate the impact of HIV infection in modulating the NK cell compartment of both HIC and non-HIC. Our findings suggest that both epigenetic and genetic factors play a role in shaping the NK cell phenotype in HIV infection. We identified a unique memory NK cell subset in PLHIV, distinct from the CMV-induced memory NK phenotype. As these memory NK cells show increased anti-viral properties in HIC, we propose that this NKG2C+ memory NK population is one of the mechanisms associated with the spontaneous control of HIV. Understanding the mechanisms behind the development and function of this HIV-specific NK cell memory, as well as its interactions with the adaptive immune system, could lead to new therapeutic strategies that harness the innate immune response for improved HIV control. These insights may not only inform the development of treatments to boost the body's natural ability to fight HIV, but also pave the way for more personalised therapy even for those not on antiretroviral therapy.

## Contributors

A.H. and A.L.G. contributed equally to this work. A.H., A.L.G., and J.C.S. conceived the study and developed the overall research strategy. A.H., A.L.G., and A.N. performed experiments and acquired data. H.J.P.M.K. and A.N. supported flow cytometry analysis and data curation. S.D.E.R. and V.M. conducted the genome-wide association study (GWAS) and data curation. A.H., A.L.G., A.N., S.D.E.R., V.M., N.V., E.T.F., A.G., W.A.J.W.V., M.J.T.B., L.E.E., J.S., M.B., R.M., A.M., C.R., A.V., and J.L. contributed to methodological development and application. A.H. and A.L.G. carried out data analysis and generated visualisations. A.J.A.M.V., M.G.N., and L.A.B.J. acquired funding and provided scientific oversight. M.C.P.J.C. coordinated project administration. A.H., A.L.G., and J.C.S. wrote the original draft of the manuscript. All authors contributed to the critical revision of the manuscript. A.H., A.L.G. and J.C.S. accessed and verified the underlying data. All authors read and approved the final version of the manuscript.

## Data sharing statement

The original contributions presented in this study are included in the article and Supplementary Materials. Additional data, including analysis code and algorithm tools used in this study can be shared by the authors upon request. Due to participant privacy concerns and consent limitations, individual-level genetic and clinical data cannot be publicly shared but can be made available under a data transfer agreement. For further information, please contact the corresponding author: jessica.dossantos@radboudumc.nl.

## Declaration of interests

MGN is a scientific founder of TTxD, Biotrip, Lemba TX, and Salvina TX. LABJ is scientific founder of TTxD, Lemba TX, and Salvina TX. AH, NV, SDER, and JCS received a “New Investigators Scholarship” to attend the *Conference on Retroviruses and Opportunistic Infections* 2024 and 2025 to present data outside of the current manuscript. The remaining authors declare that they have no competing interests.
