# Supplementary material for: Phenotypic and epigenetic profiles of circulating NK cells in spontaneous HIV-1 controllers
Source: eBioMedicine. 2025 Sep 29;120:105948. doi: 10.1016/j.ebiom.2025.105948 (PMC12513069; doi:10.1016/j.ebiom.2025.105948)
Supplement: Supplementary Tables and Figs [file mmc1.docx]

## **Supplementary Information**

**Supplementary Figures**


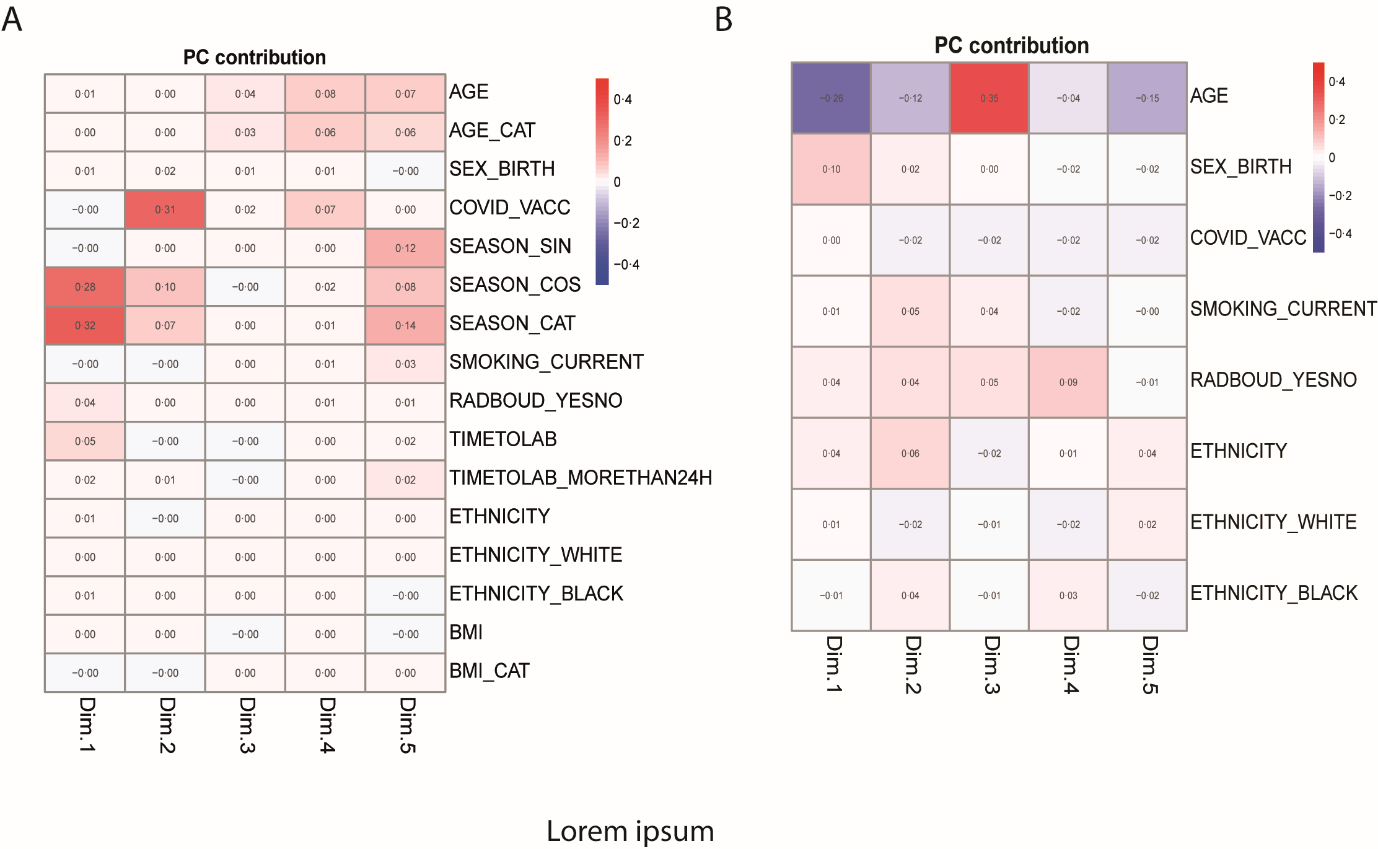


**Figure S1:** **Confounder selection for linear regression model.** (A) Principal component contribution analysis of confounders used to compare NK cell populations of the whole-blood flowcytometry analysis from the 2000HIV cohort and (B) from the 2000HIV-TRAINED Substudy with CD56+ sorted NK cells. RADBOUD_YESNO refers to whether participants were recruited at Radboud University Medical Center or not.


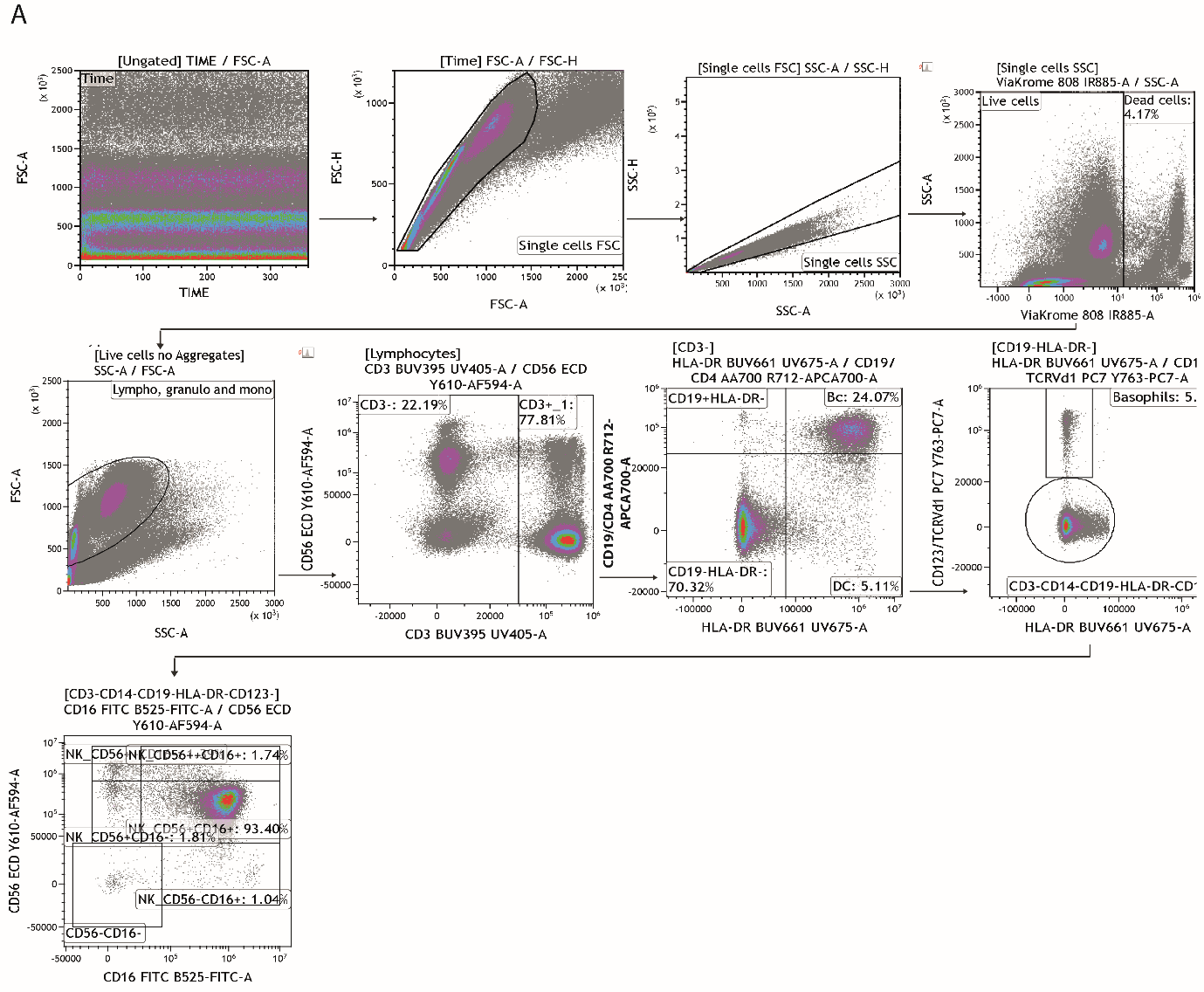


**Figure S2:** **General gating strategy for NK cells in the 2000HIV panel.** (A) Gating strategy applied to determine the percentages of CD56^bright^CD16^-^, CD56^bright^CD16^+^, CD56^dim^CD16^+^, CD56^dim^CD16^-^ and CD56^low^CD16^+^ NK cells subpopulations in whole-blood.


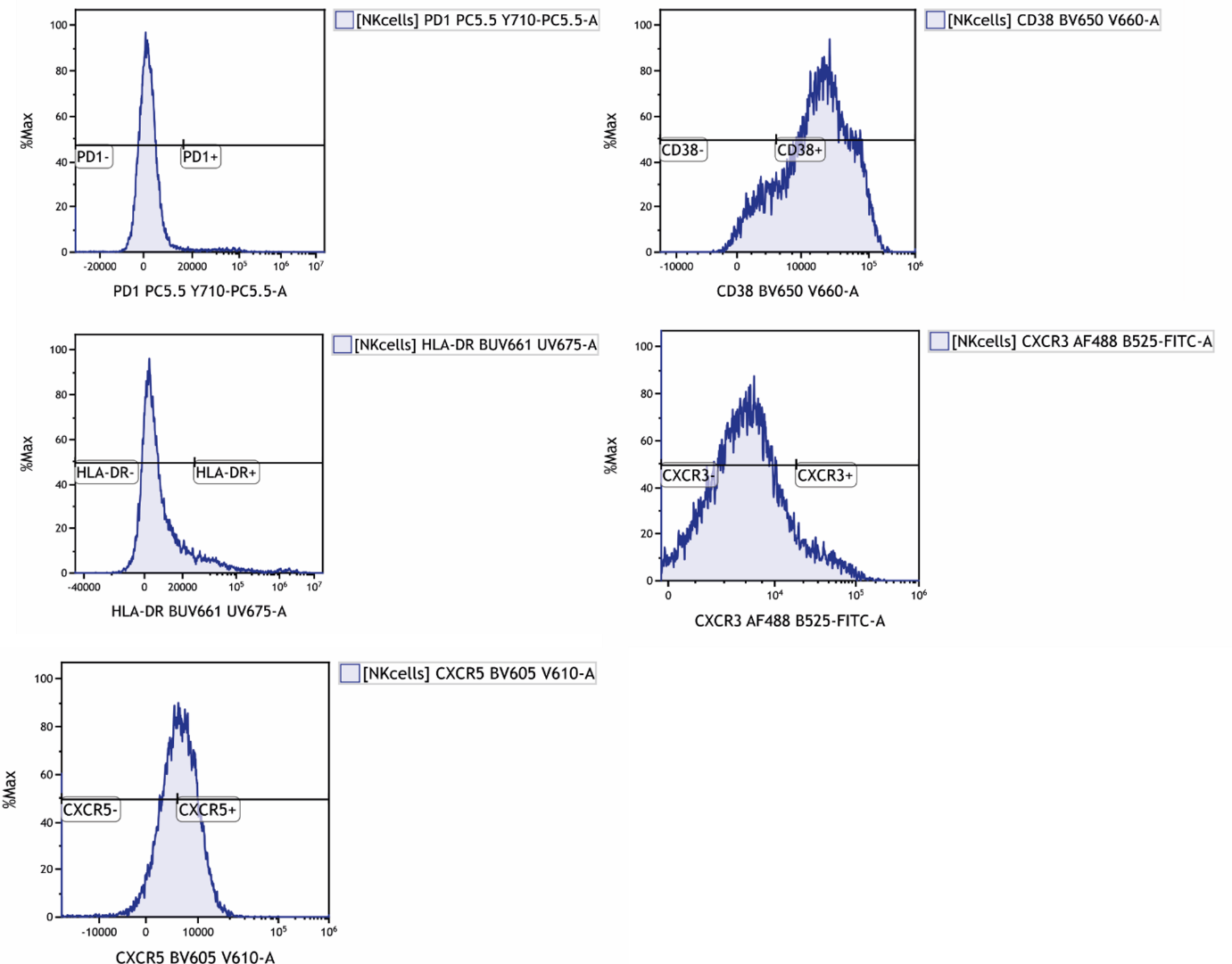


**Figure S3**: **Quantification of NK cell receptors expression in the 2000HIV panel.** Strategies used to determine the percentages of CD45^+^CD3^-^CD56^+^ live NK cells expressing chemokine receptors CXCR5, CXCR4, CXCR3, CCR7, CCR6, CCR5, CCR4, exhaustion markers PD1, CD38 and the activation marker HLA-DR in whole-blood.


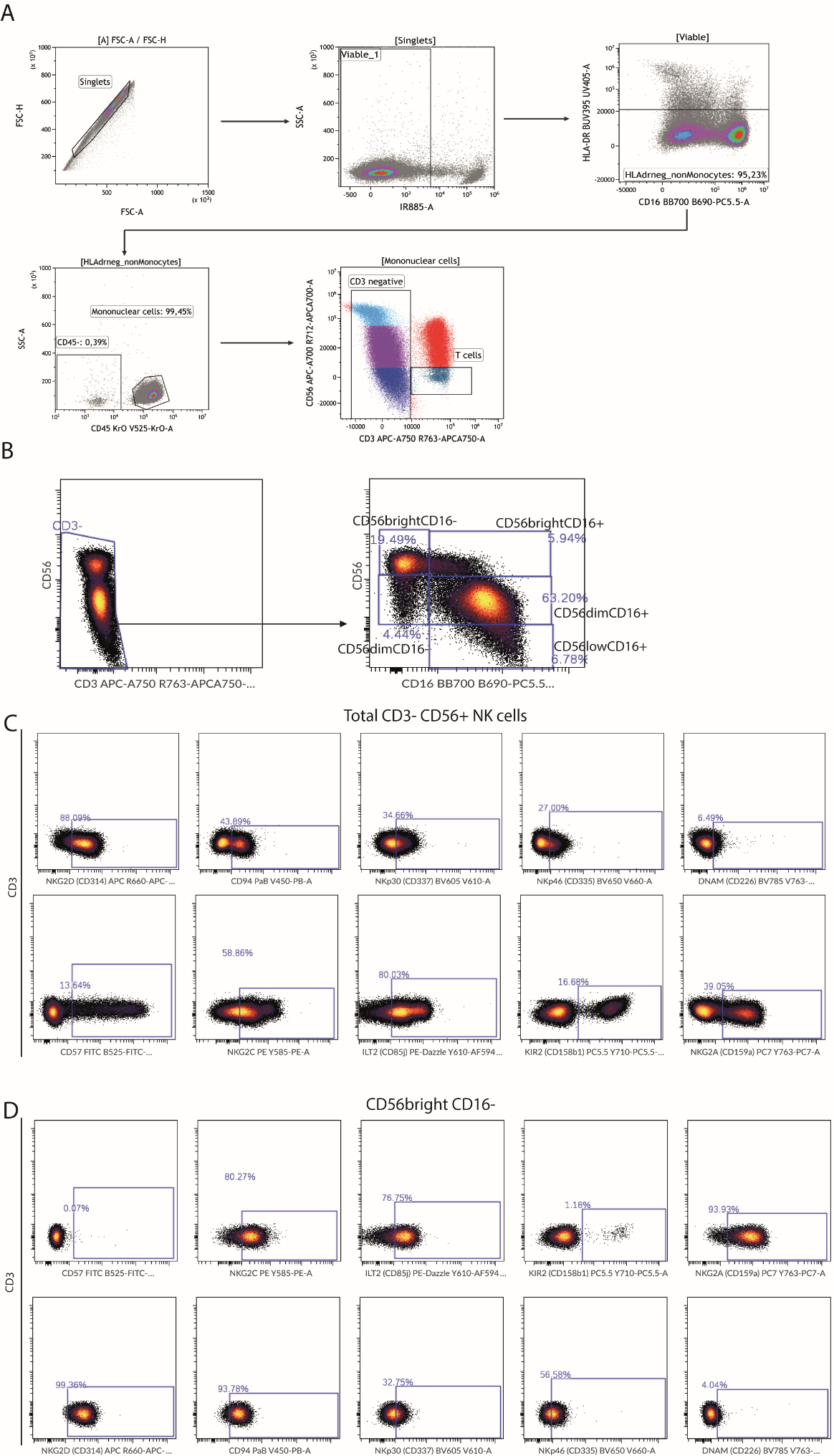


**Figure S4: Gating strategy to determine NK cell subpopulations and receptor expression in the 2000HIV-TRAINED substudy in MACS isolated NK cells.** (A) Gating strategy used in MACS-isolated NK cells in participants part of the 2000HIV-TRAINED substudy. Data from manually gated singlet/lived/mononuclear cells CD45+/CD3-/CD56+ NK cells were uploaded to the Cytobank platfrom and (B) NK cell subpopulations were gated based on their CD56 and CD16 expression. NK cell populations (total CD3-CD56+ NK cells, CD56brightCD16-, CD56brightCD16+, CD56dimCD16-, CD56dimCD16+, CD56lowCD16+) were saved as populations on the platform (C) Gating strategy of functional NK cell receptors CD57, CD94, DNAM, ILT2, KIR2DL2/3, NKG2A, NKG2C, NKG2D, NKp30 and NKp40 in Cytobank on total CD3^-^ CD56^+^ NK cells and (D) CD56^bright^CD16^-^ NK cells. Boolean gating with the CytoBank platform allowed to stratify for NK cell populations saved as population and identification of NK cell expressing functional markers. Percentages were exported from the online platform for further analysis.


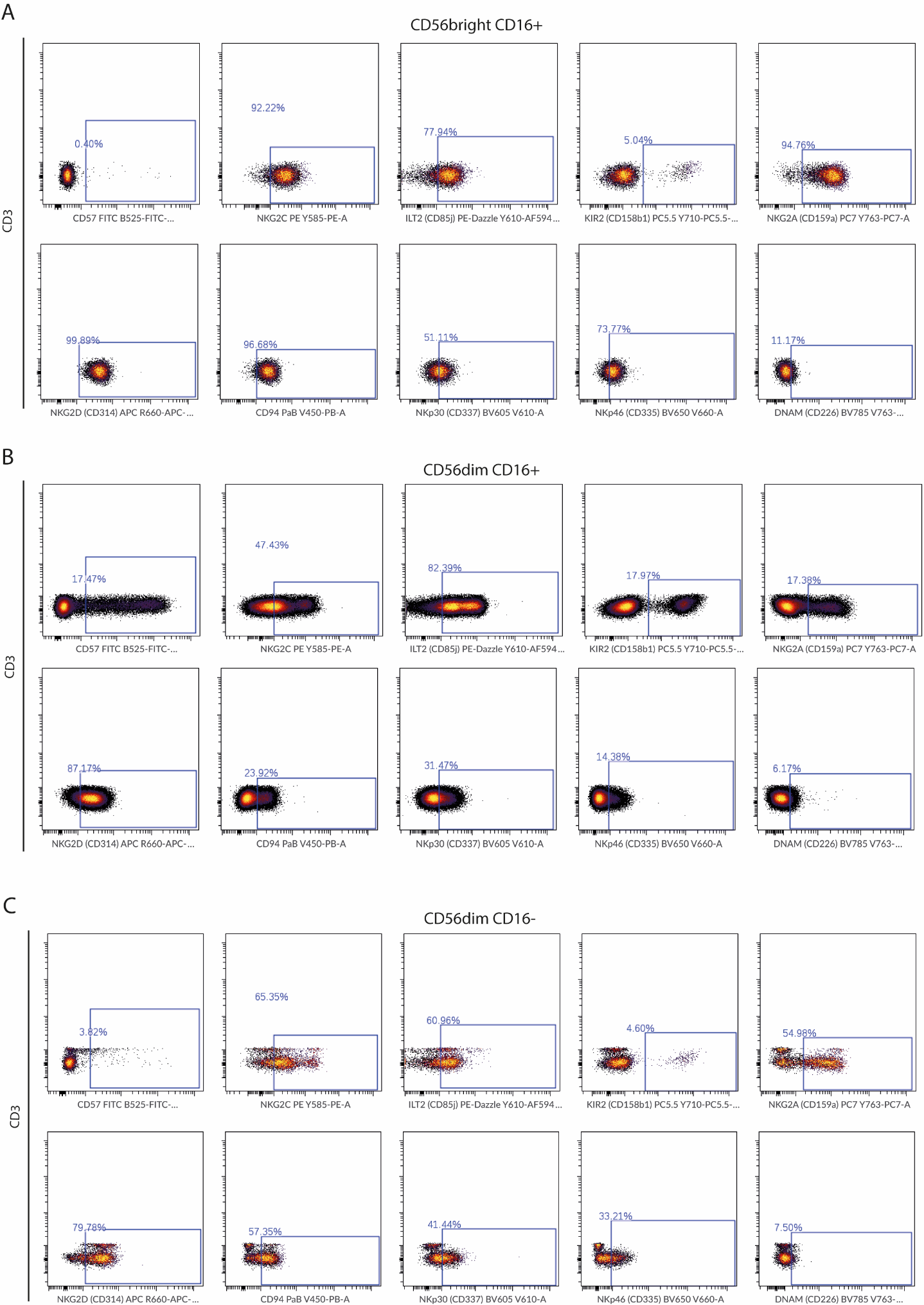


**Figure S5:** **Gating strategy to determine NK cell subpopulations and receptor expression in the 2000HIV-TRAINED substudy in MACS isolated NK cells (extended).** (A) Gating strategy of functional NK cell receptors CD57, CD94, DNAM, ILT2, KIR2DL2/3, NKG2A, NKG2C, NKG2D, NKp30 and NKp40 in Cytobank on CD56^bright^ CD16^+^ NK cells, (B) CD56^dim^CD16^+^ NK cells and (C) CD56^dim^CD16^-^ NK cells. Functional receptors on NK cell subpopulations were gated and quantified using Boolean gating strategy.


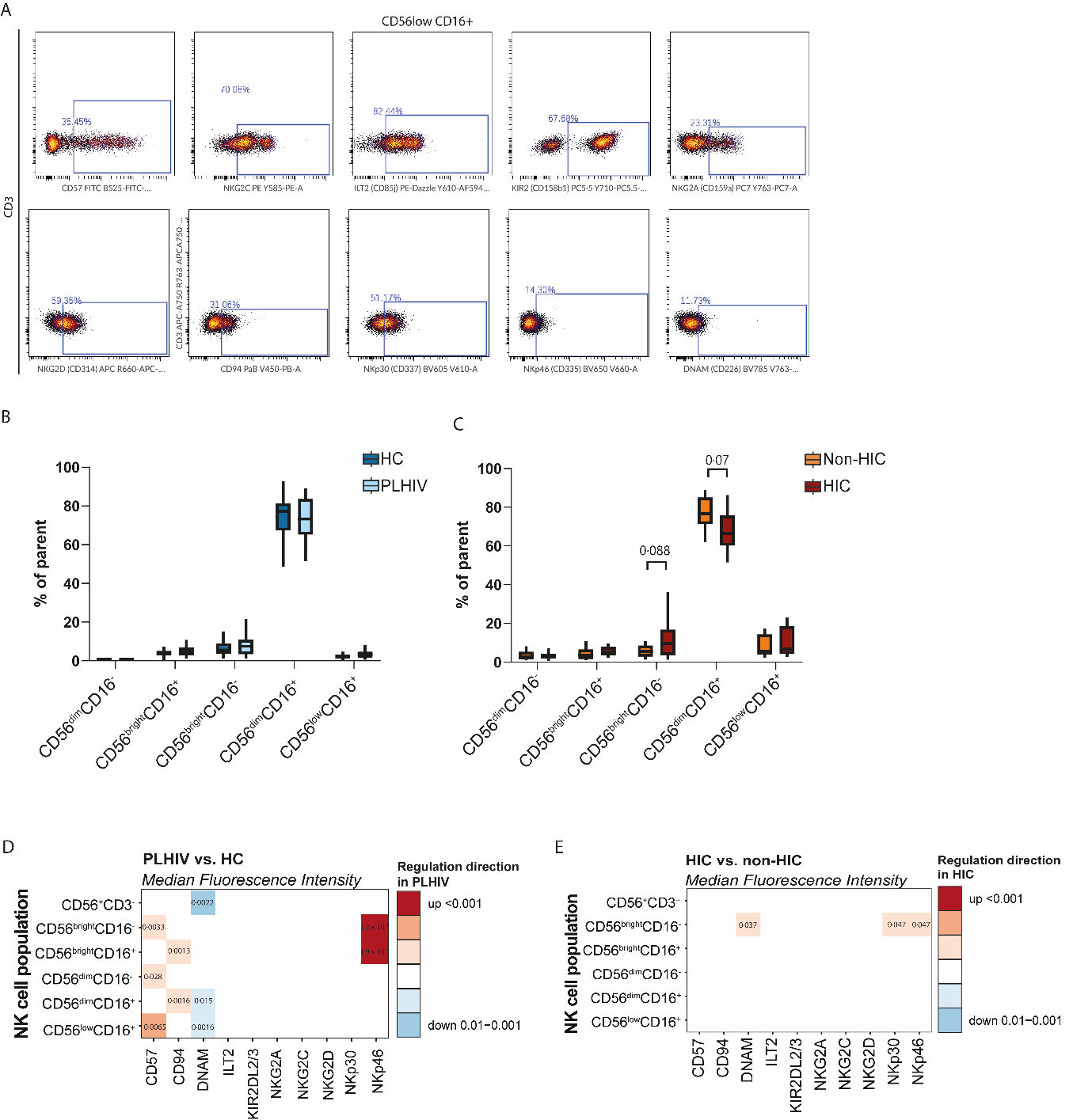


**Figure S6:** **Gating strategy to determine NK cell subpopulations and receptor expression in the 2000HIV-TRAINED substudy in MACS isolated NK cells (extended).** (A) Gating strategy of functional NK cell receptors CD57, CD94, DNAM, ILT2, KIR2DL2/3, NKG2A, NKG2C, NKG2D, NKp30 and NKp40 in Cytobank on CD56^low^CD16^+^ NK cells. Functional receptors on NK cell subpopulations were gated and quantified using Boolean gating strategy. (B) Percentages CD56^bright^CD16^-^, CD56^bright^CD16^+^, CD56^dim^CD16^+^, CD56^dim^CD16^-^ and CD56^low^CD16^+^ NK cell subpopulations assessed in CD56^+^ sorted NK cells of 23 PLHIV and 21 HC. (C) Percentages CD56^bright^CD16^-^, CD56^bright^CD16^+^, CD56^dim^CD16^+^, CD56^dim^CD16^-^ and CD56^low^CD16^+^ NK cell subpopulations assessed in CD56^+^ sorted NK cells of 13 HIC and 10 non-HIC. Statistical analysis was performed using a linear regression model, corrected for sex and age. Data are represented as median with interquartile range. Whiskers extend to 1.5 × IQR. Exact P-values are shown. (D) MFIs of total CD56+CD3-, CD56brightCD16-, CD56brightCD16+, CD56dimCD16+, CD56dimCD16- and CD56lowCD16+ NK cells subpopulations expressing CD57, CD94, DNAM, ILT2, KIR2DL2/3, NKG2A, NKG2C, NKG2D, NKp30 and NKp40 between PLHIV and HC, adjusted for sex and age as well as in (E) HIC vs. non-HIC. Data represented as estimates. Exact p-values are shown.


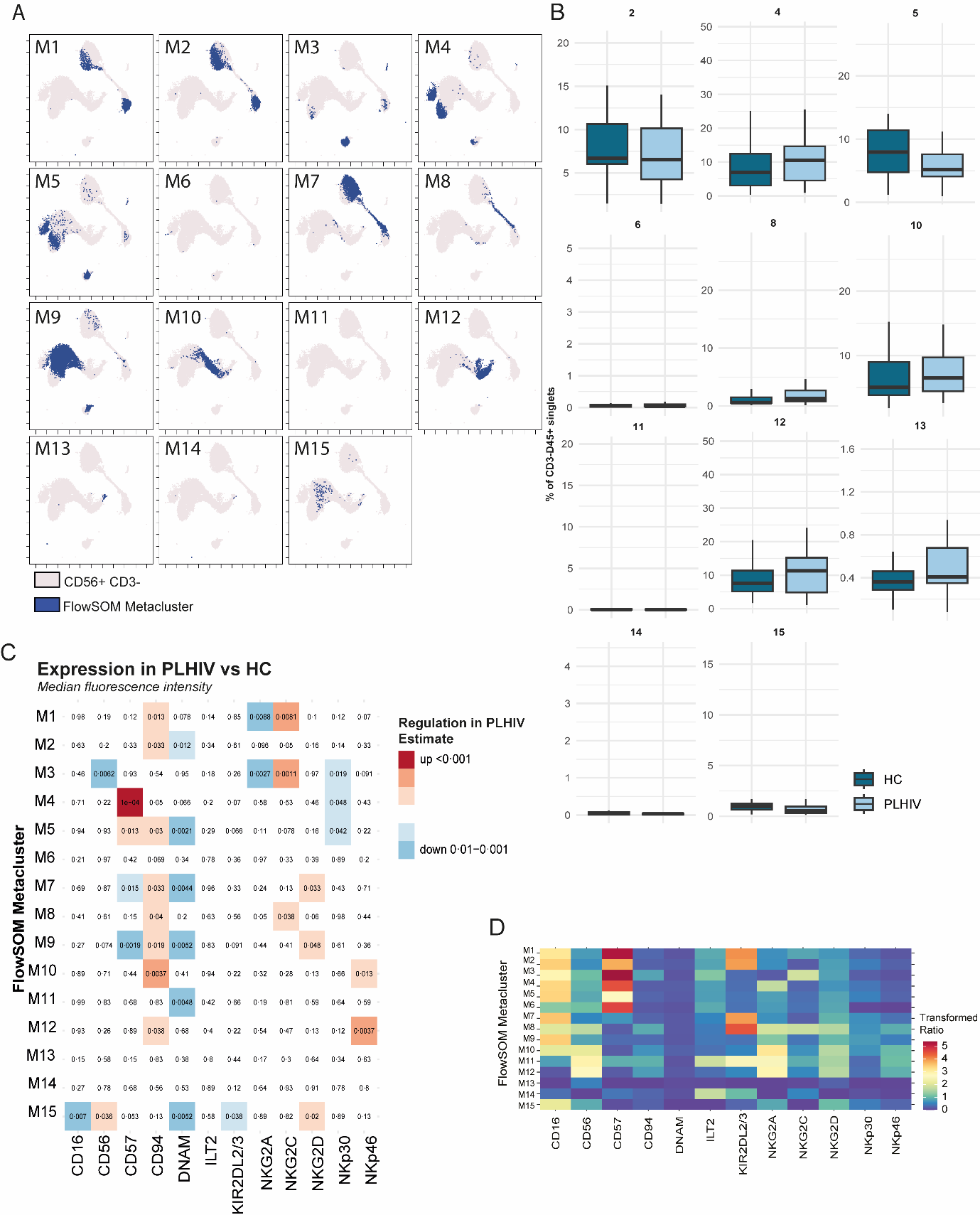


**Figure S7: Comparison of metaclusters among PLHIV and healthy controls.** (A) Overlay of FlowSOM metaclusters 1-15 over UMAP visualization of an exemplary PLHIV from 2000HIV-TRAINED substudy. (B) Percentage of CD3^-^CD45^+^ cells between PLHIV and HC in each flowSOM metacluster. Significance was tested using a Wilcoxon rank-sumtest. Data are represented as median with interquartile range. Whiskers extend to 1.5 × IQR, outliers were considered to be beyond those limits. Exact P-values are shown. (C) NK cell marker expression between PLHIV and HC within each FlowSOM metacluster. (D) Representation of marker expression among FlowSOM metaclusters. The transformed ratio was calculated using the column's minimum of the cumulative median.


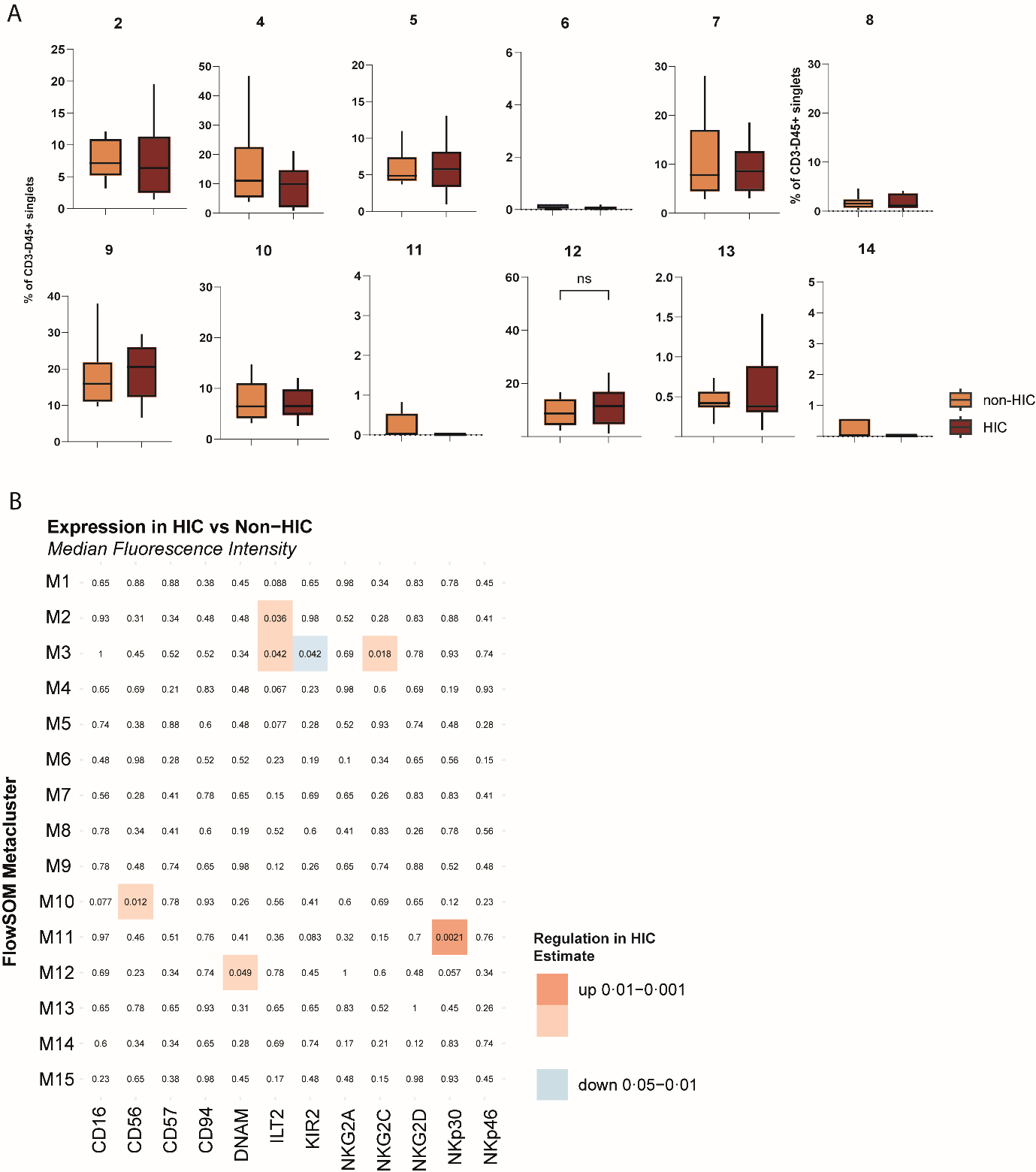


**Figure S8: Comparison of metaclusters among HIC and non-HIC.** (A) Percentage of CD3^-^CD45^+^CD56^+^ cells between HIC and non-HIC within all flowSOM metaclusters. (B) NK cell marker expression between HIC and non-HIC within each FlowSOM metacluster. Significance was tested using a Wilcoxon rank-sum test. Data are represented as median with interquartile range. Whiskers extend to 1.5 × IQR. Exact P-values are shown.


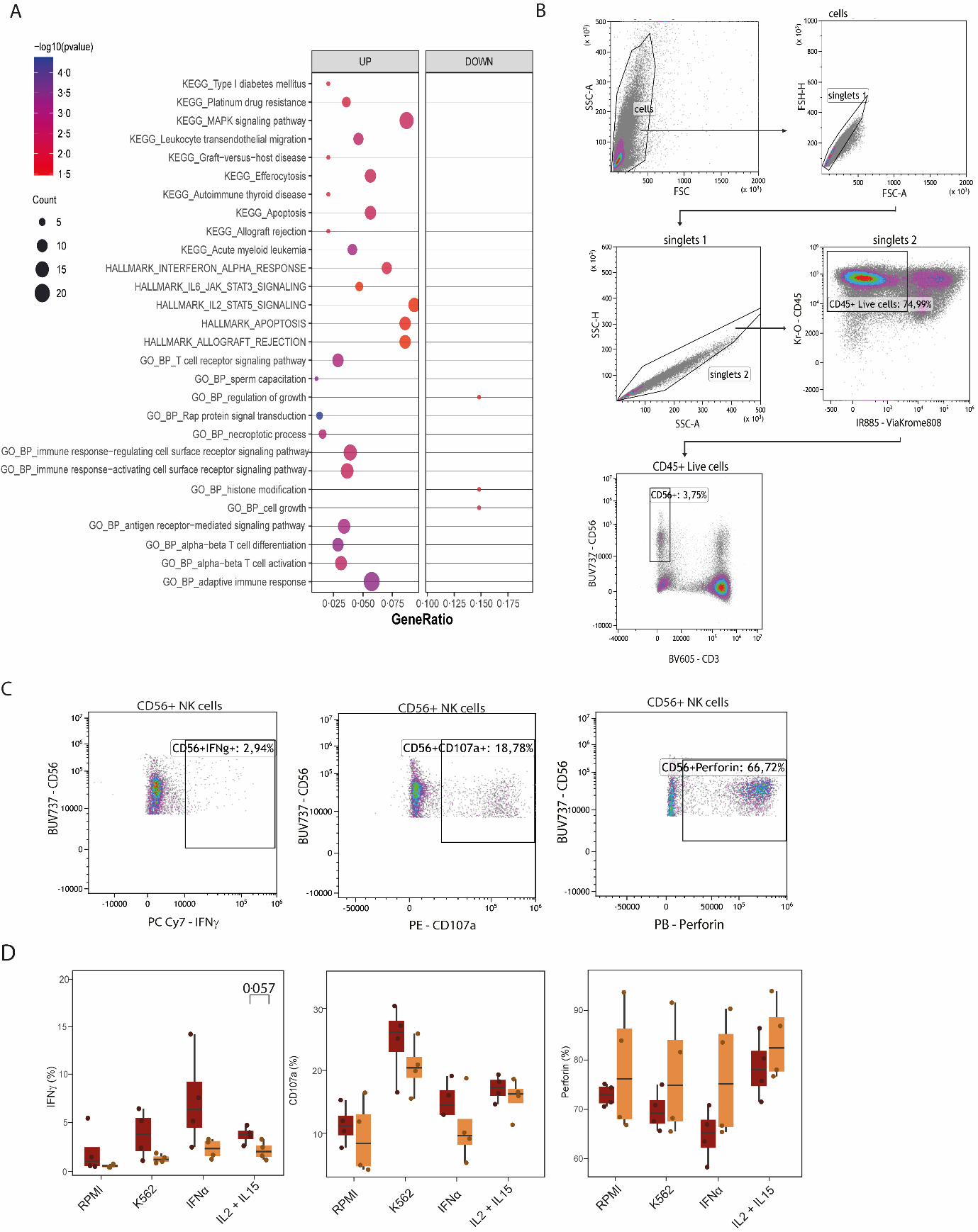


**Figure S9: Epigenetic modifications and functional assessment of NK cells of HIC and non-HIC.** (A) Functional enrichment of up- and down-regulated H3K4me3-enriched regions measured in NK cells of HIC compared to non-HIC using the Hallmark database using Hallmark, KEGG, GO-BP databases(B) Gating strategy for NK cells among PBMCs (C) Gating for NK cell effector molecules CD107a, IFNγ, Granzyme B and Perforin in CD3^-^CD56NK cells. (D) IFNγ, CD107a, and Perforin expression by CD56^+^ NK cells of 4 HIC, 4 non-HIC. PBMCs of HIC and non-HIC were activated for 10 hours with IL-2 and IL-15 or left unstimulated (RPMI) and afterward co-cultured with either MHC-deficient K562 cell lines for 4 hours or stimulated with IFNα. Cells were stained with appropriate antibodies, and IFNγ production, Perfroin production and CD107a expression were measured via flowcytometry. Data are represented as median with interquartile range. Whiskers extend to 1.5 × IQR. Significance was tested using a Wilcoxon rank-sum test. Exact p-values are shown.


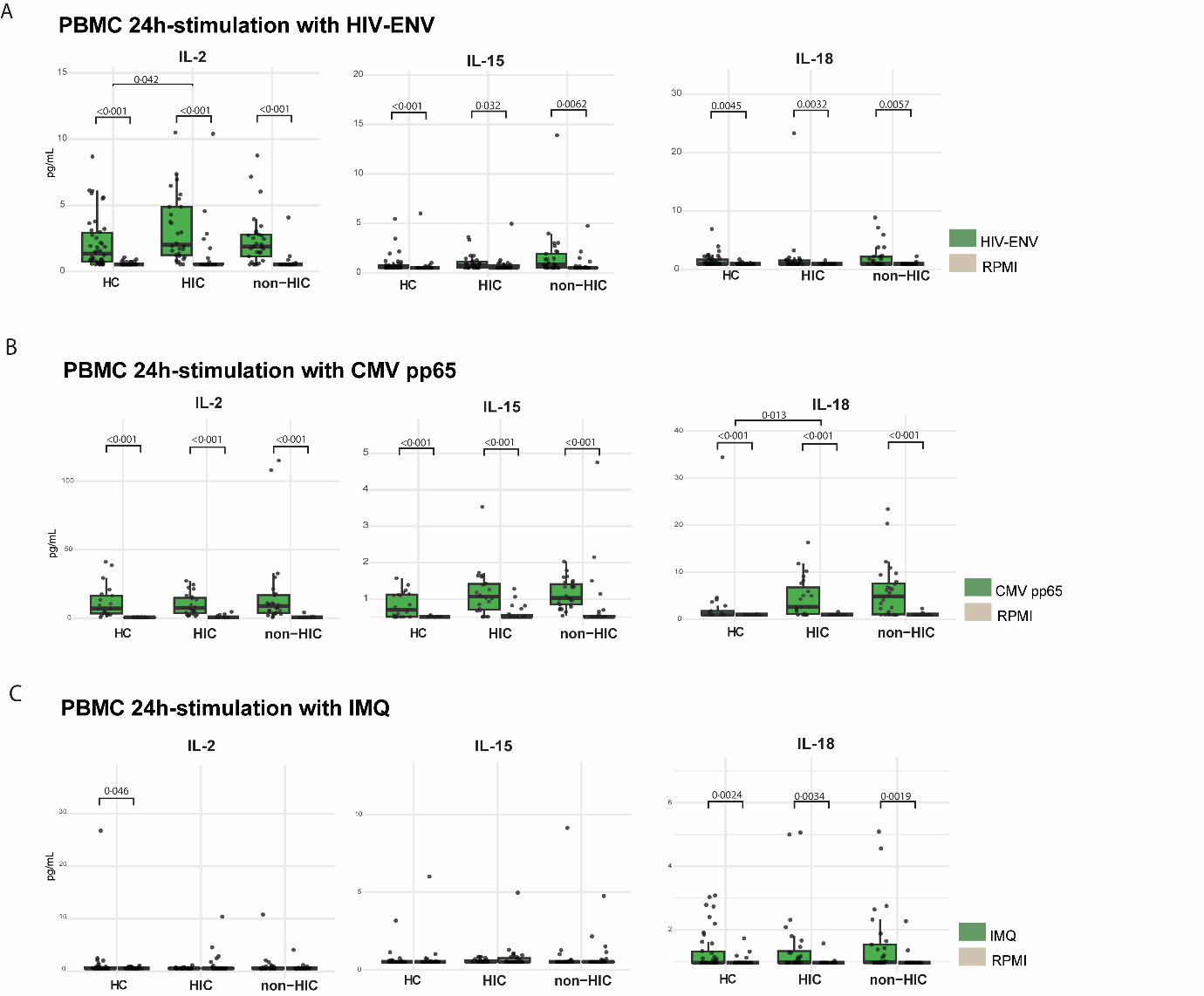


**Figure S10: Ex-vivo production of NK cell stimulating cytokines of PBMCs stimulated with varoíous viral peptides**Ex-vivo cytokine production of PBMCs of HIC, non-HIC and healthy controls (HC) upon stimulation with RPMI (negative control) or (A) HIV-ENV, (B) CMVpp65 or (C) IMQ. Data are represented as median with interquartile range. Significance between stimuli was tested using a Sign test and between participant groups using a Wilcoxon rank-sum test. Data are represented as median with interquartile range. Whiskers extend to 1.5 × IQR. Exact P-values are shown.


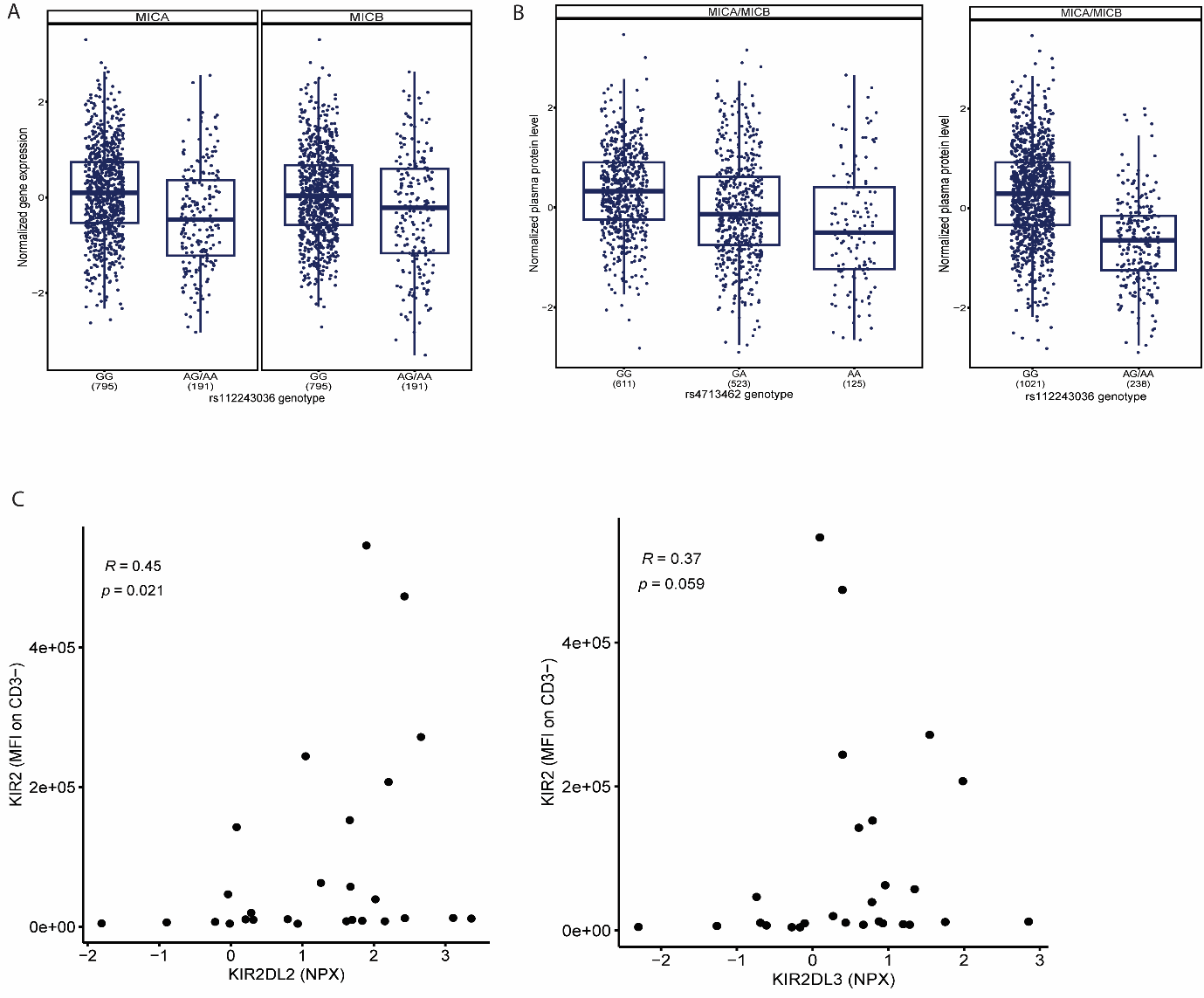


**Figure S11: eQTL and pQTL results of identified SNPs associated with HIV control and KIR2DL2/3 are also associated with MICA and MICB.**  (A) eQTL analysis based on the SNP rs112243036 regulating the gene expression of the NK cell ligands MICA and MICB . (B) pQTL analysis of the top SNPs rs112243036 and rs4713462 regulating the protein levels of MICA and MICB. Data are represented as median with interquartile range. Whiskers extend to 1.5 × IQR. (C) Spearman correlation between plasma levels of KIR2DL2 and KIR2DL3 with respective MFI levels of KIR2DL2/3 on CD3-CD56+ NK cells.

**Supplementary Tables**

**Table S1**: Manufacturer information of drop-in antibodies used for whole-blood NK cell staining.

| Panel | Antibody | Clone | Company | Cat # | RRID |
| --- | --- | --- | --- | --- | --- |
| Panel 1 | HLA-DR-BUV661 | G46-6 | BD Biosciences | 612981 | AB_2916889 |
|  | CD3-BUV395 | UCHT | BD Biosciences | 563546 | AB_2744387 |
|  | ViaKrome 808 |  | Beckman Coulter | C36628 | NA |
| Panel 2 | CD38-BV650 | HB-7 | BioLegend | 569966 | AB_3685439 |
|  | CD4-AF594 | RPA-T4 | BioLegend | 300544 | NA |
|  | CXCR5-BV605 | J252D4 | BioLegend | 356930 | AB_2566227 |
|  | ViaKrome 808 |  | Beckman Coulter | C36628 | NA |

**Table S2:** Pre-coated antibodies in custom-made DuraClone tubes (Beckmann Coulter, Brea, California, USA) of Panel 1 and Panel 2 used for whole-blood NK cell staining.

| Panel | Antibody |
| --- | --- |
| Panel 1 | CD45-KrO |
|  | CD16-FITC |
|  | CD56-ECD |
|  | CD19-AA700 |
|  | CD123-PC7 |
| Panel 2 | CD45-KrO |
|  | CXCR3-AF488 |
|  | PD1-PC5·5 |
